# Evaluation of mobile health applications using the RE-AIM model: systematic review and meta-analysis

**DOI:** 10.3389/fpubh.2025.1611789

**Published:** 2025-08-04

**Authors:** Emanuele Louise Gomes de Magalhães Jorge, Emily de Souza Ferreira, Michele Duarte Pereira, Ana Luiza Paes Mingote, João Henrique Corrêa Silva, Tiago Ricardo Moreira, Rosângela Minardi Mitre Cotta

**Affiliations:** ^1^Department of Nutrition and Health, Federal University of Viçosa, Viçosa, Brazil; ^2^Department of Medicine and Nursing, Federal University of Viçosa, Viçosa, Brazil

**Keywords:** mobile application, digital intervention, health promotion, re-aim, digital health

## Abstract

**Background:**

The Reach, Effectiveness, Adoption, Implementation, and Maintenance (RE-AIM) model has been used as an instrument to determine the impact of the intervention on health in digital format. This study aims to evaluate, through a systematic review and meta-analysis, the dimensions of RE-AIM in interventions carried out by mobile health apps.

**Methods:**

The systematic review and meta-analysis were conducted following the Preferred Reporting Items for Systematic Reviews and Meta-Analyses (PRISMA) guidelines and involved searching six databases - Medline/PubMed, Embase, CINAHL, Virtual Library in Health, and Cochrane Library. The review included randomized, cross-sectional, and cohort clinical trials assessing the prevalence of each RE-AIM dimension according to the duration of the intervention in days. The quality of the selected studies was evaluated using the Joanna Briggs Institute tool. The random effects meta-analysis method was used to explain the distribution of effects between the studies, by Stata® software (version 11.0) and publication bias was examined by visual inspection of graphs and Egger’s test.

**Results:**

After analyzing the articles found in the databases, and respecting the PRISMA criteria, 21 studies were included, published between 2011 and 2023 in 11 countries. Improvements in health care and self-management were reported for various conditions. The result of the meta-analysis showed a prevalence of 67% (CI: 53–80) for the reach dimension, of 52% (CI: 32–72) for effectiveness, 70% (CI: 58–82) for adoption, 68% (CI: 57–79) for implementation and 64% (CI: 48–80) for maintenance.

**Conclusion:**

The RE-AIM dimensions are useful for assessing how digital health interventions have been implemented and reported in the literature. By highlighting the strengths and areas requiring improvement, the study provides important input for the future development of mobile health applications capable of achieving better clinical and health promotion outcomes.

**Systematic Review Registration:**

PROSPERO. International Prospective Registry of Systematic Reviews. CRD42024556886.

## Introduction

The increase in chronic non-communicable diseases (CNCDs) worldwide has led to the rapid expansion of digital mobile health (mHealth). With the increasing use of mobile devices, digital health applications are emerging as promising tools to expand access to the monitoring and managing of these conditions, enabling real-time support and adherence to safe practices that promote behavioral changes essential for the prevention and control of NCDs (1).

Digital health has been advanced by Information and Communication Technologies (ICTs), which enable the transmission of information through digital means, such as computers and wireless networks. These technologies were first introduced in the healthcare sector in the United States in the 1970s (2, 3) and have since been adopted in numerous countries worldwide. With the onset of the COVID-19 pandemic in 2020, there was greater incorporation of these technologies, both in public and private services, for treatment and monitoring of health care adapted to the context of social isolation, leading to a global expansion of the use of online health services for medical consultations and training of health professionals (4).

The literature has shown how the use of ICTs in health services is an excellent tool to ease the flow and, above all, to be able to serve a greater number of people in a universal and equal way. Healthcare applications can be used to optimize results and reduce health risks, as well as to understand the determinants of health promotion and disease (5–7).

Enabled by digital technologies, mobile applications are widely accessible to individuals across various settings, applicable at multiple levels of care -primary, secondary, and tertiary- and have demonstrated a positive impact on health outcomes (8).

In addition, they can be applied in real-life scenarios to provide continuous monitoring and management of health and/or disease parameters (8), on an individual and group basis, encouraging healthy behaviors, preventing or reducing health problems, supporting self-management of chronic diseases, improving users’ knowledge, reducing the number of medical consultations and providing opportunities for different areas of health to develop remote care (9–11).

The implementation of digital health intervention programs has emerged as a key strategy in public health to enhance patient support and improve access to various interventions aimed at health promotion and disease prevention. These programs facilitate treatment adherence and integration while promoting behavioral strategies through messaging, goal tracking, and encouragement of behavior change. By improving individual health outcomes, they contribute to the delivery of high-quality care by healthcare professionals (4, 12).

In this context of digital interventions, the Reach, Effectiveness, Adoption, Implementation, and Maintenance (RE-AIM) model has been used as a tool to help improve the chances of implementing and, above all, planning, monitoring, and evaluating intervention programs in practices and policies focused on promoting healthy habits (13, 14). The RE-AIM framework is made up of five dimensions, translated and adapted from the acronyms: Reach, Effectiveness, Adoption, Implementation, and Maintenance, which together determine the impact on health.

The first study to apply RE-AIM was published in 1999 and since then many researchers and evaluators have used this tool to check what is working and what is not in their programs and use this information to plan quality improvement activities, as well as the long-term sustainability of intervention programs (15–17). Despite its widespread use, there is still no consensus in the literature on the quality of the RE-AIM model for evaluating digital health intervention programs. To this end, this study aims to evaluate mobile health applications using the dimensions of the RE-AIM model, through a systematic review and meta-analysis.

## Methodology

### Study design and search strategy

This is a systematic review and meta-analysis, conducted following the Preferred Reporting Items for Systematic Reviews and Meta-Analyses (PRISMA) guidelines (18) and registered with the International Prospective Register of Systematic Reviews (PROSPERO), under protocol number CRD4202455688.

The Population, Intervention, Comparator, Outcomes, and Studies (PICOS) method guided the establishment of the inclusion criteria, based on the following guiding question: “What are the scope, effectiveness, adoption, implementation, and maintenance (RE-AIM dimensions) of interventions carried out by mobile health apps?.” The problem considered was mHealth apps, the intervention was the use of the RE-AIM model to evaluate mHealth apps, and there was no comparison. The outcome considered was the RE-AIM dimensions (reach, efficacy, adoption, implementation, and maintenance), and the studies included were cohort, case–control, cross-sectional, and randomized.

The search strategy was developed using keywords from the literature, including previous reviews. Based on these terms, we analyzed which were appropriate among those established by the MeSH (Medical Subject Headings) terms, defining the following terms: Digital Health, Telehealth, Health Program, Health Intervention, and Intervention. To take into account the digital health perspective, the search terms were used in different combinations: (“RE-AIM” AND (“mobile phone” OR “digital health” OR telehealth)); (“RE-AIM” AND (“mobile phone” OR “digital health” OR telehealth) AND “intervention”); (“RE-AIM” AND (“mobile phone” OR “digital health” OR telehealth) AND (“health program” OR “health intervention”)) (Supplementary material—Appendix I).

### Data sources

The search was carried out in July 2024, in the electronic databases Medical Literature Analysis and Retrieval System Online (MEDLINE/PubMed), Scopus, Virtual Health Library (VHL), Excerpta Medica dataBASE (EMBASE), and Cumulative Index to Nursing and Allied Health Literature (Cinahls).

The bibliographic software Rayyan (Qatar Computing Research Institute, Qatar) was used to manage the references. No language or publication date filters were used in the search stage.

### Eligibility criteria

Only studies that focused on the use of mobile applications in healthcare and the RE-AIM model were included. Review articles, letters, editorials, conference proceedings, commentaries, reports, study protocols, pilot studies, case reports, and experience reports were excluded from this study. There were no date or language restrictions.

### Study selection

The selection of studies was conducted independently by three reviewers (ELGMJ, MDP, ALPM) using Rayyan software. Screening of the title, abstract, and text began, and any discrepancies were discussed with another reviewer (JHCS) to resolve them. Each of the three reviewers selected studies for possible inclusion based on the title and content of the abstract. The studies that met the inclusion criteria were analyzed in the full-text review. A reverse search was carried out, analyzing the references of the selected articles to identify any additional relevant studies not captured in the initial search.

### Data extraction

A Microsoft Excel spreadsheet (Microsoft Corp., Washington, USA) was used to organize the extracted data. The following data was collected: title, year of publication, authors, country, year of study, study design, app name, sample size, study duration, sample characterization, main intervention, main features of the mobile app, program data, results, and main conclusions.

To extract the data on the dimensions of RE-AIM, we used a table adapted from Burke et al. (19), which includes the five dimensions and the items that make up each one. We considered the percentage of adequacy, concerning adequately reported interventions, and the percentage of inadequacy, in relation to inadequately reported interventions.

### Meta-analysis

The primary outcome was the prevalence of Reach, Effectiveness, Adoption, Implementation, and Maintenance according to the duration (in days) of the intervention. The meta-analysis was performed using Stata® software (version 11.0), utilizing the Metaprop and Metareg commands. The random effect model was used to carry out the meta-analysis. Heterogeneity was assessed using the chi-squared test (χ2) with a significance level of 90% (*p* < 0.10), and its magnitude was determined by the I-squared (I2) (20).

Heterogeneity was classified as low, moderate, or high when I2 values were above 25, 50 and 75%, respectively. Heterogeneity was examined through meta-regressions using the Knapp and Hartung test (21). A univariate analysis was carried out with the following variables: year of publication, duration of the intervention and region or continent of the study (to include). A significance level of 5% was established. The presence of small-study effects was evaluated through visual inspection of the funnel plot and Egger’s test.

### Quality and risk of bias assessment

The quality of each study included in the review was assessed using the critical appraisal tool recommended by the Joanna Briggs Institute (22), specific to each type of study, and conducted independently by three researchers. The evaluation is based on a checklist with 13 questions for randomized studies, 11 for cohort studies, and eight for cross-sectional studies. Each question allows answers of yes, no, unclear, or not applicable. The results were measured as a percentage, and each item on the checklist was given the following score: 1 point for “yes” and 0 for “no” “unclear” or “not applicable.” Studies of good quality were those that scored above 75% (23).

Publication bias was examined by visual inspection of funnel plots and Egger’s test through the meta-analysis carried out using Stata® software (version 11.0). The risk of bias was also analyzed using RevMan (version 5.4). The statistical significance of the overall effect size of using the RE-AIM mobile health app assessment was determined by the 95% confidence interval (CI).

## Results

### Identification of studies

**Figure 1:**
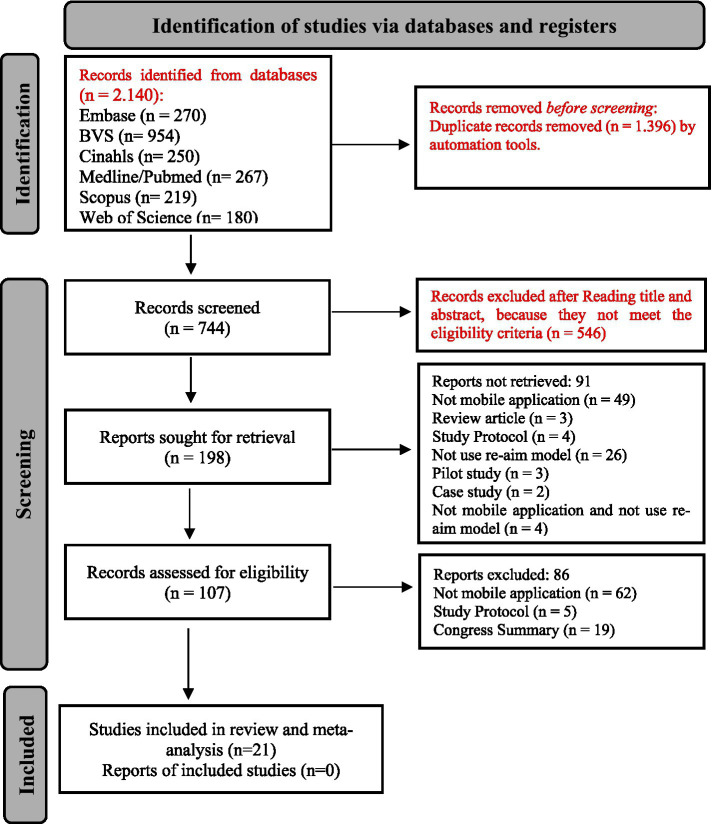
Selection flowchart of papers included in the systematic review.

The search across six databases yielded 2,140 articles. After duplicate removal, 744 articles remained and were screened based on their titles and abstracts, resulting in 198 articles. Following the application of eligibility criteria, 107 articles were retained for full-text review. A thorough assessment of the full texts ultimately led to the selection of 21 articles for inclusion in this systematic review and meta-analysis. No additional studies were identified in the reverse search. This process is shown in Figure 1.

### Characteristics of the studies included

The studies were carried out between 2011 and 2022 in different countries: eight in the United States, followed by two in China, two in the Netherlands, two in Uganda, and the rest in Canada, Switzerland, Norway, the United Kingdom, Australia, Zimbabwe and Singapore. A total of 14,823 people were assessed in the 21 studies, with a minimum sample size of 35 and a maximum of 2,961. One study evaluated only adolescents (24), one included adolescent athletes (over 12 years old) in its sample (25), one included patients aged 15 and over with sickle cell anemia, and two included patients over 15 years old who lived in a fishing community (26, 27). The other studies assessed adults and the older adult.

Different interventions were carried out on the following topics: medication use in sickle cell anemia, postectomy programs, reduction of ankle injuries, psychological intervention in chronic pain, management of low back pain, HIV prevention, adherence to antiretroviral treatment, reduction of alcohol consumption, treatment of drug addicts, nutritional monitoring of hospitalized patients, cardiac rehabilitation, health of stroke patients, healthy lifestyle for diabetes patients, promotion of physical activity, reduction of sedentary lifestyles in the workplace, training parents in pediatric care, quality of life and mental health in cancer patients and mental health of university students. The minimum intervention time was 21 days (28) and the maximum was 1,185 days (26, 27). Only one study did not provide an exact intervention time, as it varied according to the length of time patients were hospitalized (29) (Supplementary material—Appendix II).

The results of the studies showed that the use of mobile apps increases adherence to treatments, helps in managing health conditions, preventing diseases, adopting a healthy lifestyle, reducing injuries, controlling pain, and self-monitoring health. These tools were deemed effective and cost-efficient for reaching a broader population. Logistical advantages included the ability to customize app functions to individual needs, enable simultaneous use by healthcare teams, and maintain functionality offline. These features optimize healthcare professionals’ actions and extend coverage, even in areas with limited internet connectivity. Some challenges were reported, such as technical problems and difficulties in using the app, especially for older people and those with low levels of education; the issue of social stigma in cases of diseases such as HIV; the low adherence of some health professionals; and the decline in effectiveness over time, showing the difficulties of sustainability in digital health interventions (Supplementary material—Appendix II).

### Assessment of RE-AIM dimensions and items

The 21 studies were evaluated based on the suitability of the five RE-AIM dimensions -reach, effectiveness, adoption, implementation, and maintenance- and demonstrated an overall adequacy of 88.15%. According to the model proposed by Burke et al. (19), each item in these dimensions was assessed (Table 1). It is important to highlight that the RE-AIM dimensions assessed in this review reflect the reporting adequacy and presence of each dimension within the included studies, according to the authors’ own definitions and criteria, and do not directly represent clinical effectiveness or health outcomes achieved by the interventions themselves. For the “reach” dimension, the percentage of adequacy was 76.16%, with 19 studies (90.44%) meeting the “participation rate” item. For the “effectiveness” dimension, 76.16% of the studies showed adequacy, with the majority of studies meeting the “primary outcome measure” (90.44%) and “broader outcome measures” (85.68%) items.

**Table 1:** Inclusion of RE-AIM items across all interventions (*N* = 21).

RE-AIM Dimension and Items	% (*n*) (adequate)	Interventions (adequately reported)	% (*n*) (inadequate)	Interventions (inadequately reported)
Reach	76,16%			
1. Exclusion criteria	61,88 (13)	1, 2, 4–6, 8, 9, 11, 13, 14, 17, 18, 20	38,08 (8)	3, 7, 10, 12, 15, 16, 19, 21
2. Participation rate	90,44 (19)	1–9, 11–17, 19–21	9,52 (2)	10, 18
3. Representativeness	85,68 (18)	1–11, 13–17, 19, 21	14,28 (3)	12, 18, 20
4. Use of qualitative methods to understand reach and/or recruitment	66,64 (14)	1–5, 8, 10, 13, 15–17, 19–21	33,32 (7)	6, 7, 9, 11, 12, 14, 18
Effectiveness	76,16%			
5. Measure of primary outcome	90,44 (19)	1–15, 17, 18, 20, 21	9,52 (2)	16, 19
6. Measure of broader outcomes (i.e., QoL, negative outcomes)	85,68 (18)	1–10, 13–16, 18–21	14,28 (3)	11, 12, 17
7. Measure of robustness across subgroups	57,12 (12)	1–8, 10, 17, 18, 19	42,84 (9)	9, 11–16, 20, 21
8. Measure of short-term attrition	71,40 (15)	2–7, 9, 12, 13, 15, 16, 18–21	28,56 (6)	1, 8, 10, 11, 14, 17
9. Use of qualitative methods/data to understand outcomes	76,16 (16)	1–6, 8, 10, 13–17, 19–21	23,80 (5)	7, 9, 11, 12, 18
Adoption-Setting	67,83%			
10. Setting exclusions	66,64 (14)	1–3, 6–11, 13, 14, 18, 19, 21	33,32 (7)	4, 5, 12, 15–17, 20
11. Setting adoption rate	85,68 (18)	1–5, 7, 8, 11–21	14,28 (3)	6, 9, 10
12. Setting representativeness	42,84 (9)	1–6, 10, 16, 19	57,12 (12)	7–9, 11–17, 18, 20, 21
13. Use of qualitative methods to understand adoption at setting level	76,16 (16)	1–6, 8, 10, 11, 13–15, 17, 19–21	23,80 (5)	7, 9, 12, 16, 18
Adoption-Staff	63,07%			
14. Staff exclusions	52,36 (11)	1, 3, 5–7, 10, 13, 14, 18, 19, 21	47,60 (10)	2, 4, 8, 9, 11, 12, 15–17, 20
15. Staff participation rate	66,64 (14)	1, 3–7, 10, 12, 13, 16, 17, 19–21	33,32 (7)	2, 8, 9, 11, 14, 15, 18
16. Staff representativeness	71,40 (15)	1, 3, 5–7, 10–16, 19–21	28,56 (6)	2, 4, 8, 9, 17, 18
17. Use of qualitative methods to understand staff participation	61,88 (13)	1, 3, 5, 7, 8, 10, 13–15, 17, 19–21	38,08 (8)	2, 4, 6, 9, 11, 12, 16, 18
Implementation	65,68%			
18. Delivered as intended	76,16 (16)	2–11, 13, 16–20	23,80 (5)	1, 12, 14, 15, 21
19. Adaptations to intervention	80,92 (17)	1–5, 7, 8, 10–14, 16, 17, 19–21	19,04 (4)	6, 9, 15, 18
20. Cost of intervention (time or money)	38,08 (8)	1–3, 11, 14, 19–21	61,88 (13)	4–10, 12, 13, 15–18
21. Consistency of implementation across staff/ time/settings subgroups	76,16 (16)	1–4, 7, 8, 10–14, 16, 17, 19–21	23,80 (5)	5, 6, 9, 15, 18
22. Use of qualitative methods to understand implementation	57,12 (12)	3, 5, 7, 8, 10, 13–15, 17, 19–21	42,84 (9)	1, 2, 4, 6, 9, 11, 12, 16, 18
Maintenance-Individual	37,12%			
23. Measure of primary outcome at ≥6-month follow-up	42,84 (9)	1, 2, 4–6, 10, 12, 14, 19	57,12 (12)	3, 7–9, 11, 13, 15, 16–18, 20, 21
24. Measure of broader outcomes (i.e., QoL, negative outcomes) at follow-up	38,08 (8)	1, 2, 6, 10, 14, 18–20	61,88 (13)	3–7, 8, 9, 11–13, 15–17, 21
25. Measure of long-term robustness across subgroups	23,80 (5)	1, 2, 10, 12, 19	76,16 (16)	3–9, 11, 13-, 20, 21
26. Measure of long-term attrition	28,56 (6)	2, 7, 10, 17, 19, 21	71,40 (15)	1, 3–6, 8, 9, 11, 18, 20
27. Use of qualitative methods to understand long-term effects	52,36 (11)	1, 5–7, 10, 13, 14, 17, 19–21	47,60 (10)	2–4, 8, 9, 11, 12, 15, 16, 18
Maintenance-Setting	54,74%			
28. Program ongoing (≥6-month post-study funding)	52,36 (11)	1, 2, 7, 8, 10–12, 14, 19–21	47,60 (10)	3–6, 9, 13, 15, 16–18
29. Long-term program adaptations	66,64 (14)	1–3, 5, 7, 8, 10–12, 14, 17, 19–21	33,32 (7)	4, 6, 9, 13, 15, 16, 18
30. Some discussion of sustainability of business model	52,36 (11)	2, 3, 7, 10–12, 14, 16, 19–21	47,60 (10)	1, 4–6, 8, 9, 13, 15, 17, 18
31. Use of qualitative methods to understand setting-level institutionalization	47,60 (10)	3, 5, 8, 10, 12, 13, 17, 19–21	52,36 (11)	1, 2, 4, 6, 7, 9, 11, 14–16, 18
Overall RE-AIM	88,15%			

The table formatting was adapted from Burke et al. (19). RE-AIM = Reach, Effectiveness, Adoption, Implementation, Maintenance (13).

In the “adoption” dimension focused on the configuration of the intervention, 67.83% of the studies showed adequacy, with 18 (85.68%) studies meeting the “configuration exclusions” item. In the “adoption” dimension focused on the team, 63.07% of the studies showed adequacy, with 15 (71.40%) of them meeting the “team representativeness” item. In the “implementation” dimension, the studies showed 65.68% adequacy, with 17 (80.92%) of them meeting the “adaptations to the intervention” item. In the individual “maintenance” dimension, the studies were 37.12% adequate, and 11 (52.36%) studies met the item “use of qualitative methods to understand long-term effects.” In the “maintenance” dimension focused on the configuration of the intervention, the studies were 54.74% adequate, and 14 (66.64%) of them met the item “long-term program adaptations.”

A total of 31 RE-AIM items were assessed, and for the 21 studies, the average number of items reported was 19.38, with a minimum of eight and a maximum of 29 items per study.

Although the RE-AIM dimensions, particularly Effectiveness, include health outcome data reported by the original studies, it is important to clarify that the synthesis presented here reflects how these outcomes were categorized and reported according to each study’s own operational definitions and methodologies. The aggregated percentages should therefore be interpreted as a reflection of the reporting and implementation characteristics of the interventions, rather than as direct measures of clinical effectiveness or intervention success at the population level.

### Meta-analysis

**Figure 2:**
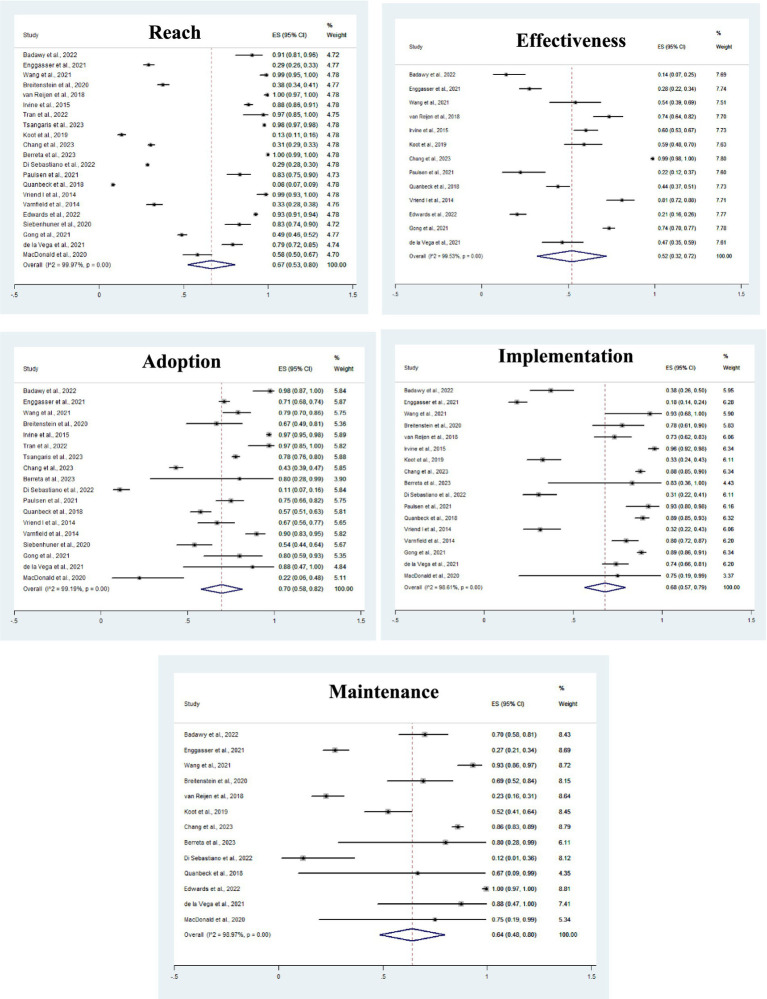
Meta-analysis of the prevalence of reach, effectiveness, adoption, implementation and maintenance, according to the days of intervention in the studies. *ES: Prevalence Estimates grouped from the studies; 95% CI: 95% Confidence Interval.

For the 21 studies analysed, the pooled prevalence estimate for Reach was 67% of the individuals assessed (CI: 53–80), for Effectiveness was 52% (CI: 32–72), for Adoption was 70% (CI: 58–82), for Implementation was 68% (CI: 57–79), and for Maintenance was 64% of the individuals present in the studies analysed (CI: 48–80) (Figure 2).

The studies showed significant heterogeneity (*p* < 0.001), considered high according to the I^2^ statistic (<98%), which can be explained by the different interventions and the diversity of the sample, which varied in age (adolescents, adults and the older adult), gender (three studies only considered males), and health conditions (morbidities such as sickle cell anemia, diabetes, cancer, chronic muscle damage and drug addiction, sedentary lifestyle, and hospitalization). Subgroup analyses were carried out for the variables year of publication, duration of intervention and region or continent of the study, but none of them showed statistical significance in any of the models analysed. Other variables of interest, such as age group and gender, were not included in the regression model because there was no complete data in the studies evaluated.

### Quality of the studies

Among the 21 articles included in this systematic review and meta-analysis, 11 met the criteria for classification as exceeding 75% based on the evaluation criteria of the Joanna Briggs Institute forms, while the remaining articles scored between 50 and 75%. None of the studies were classified as low quality.

#### Risk of bias

**Figure 3:**
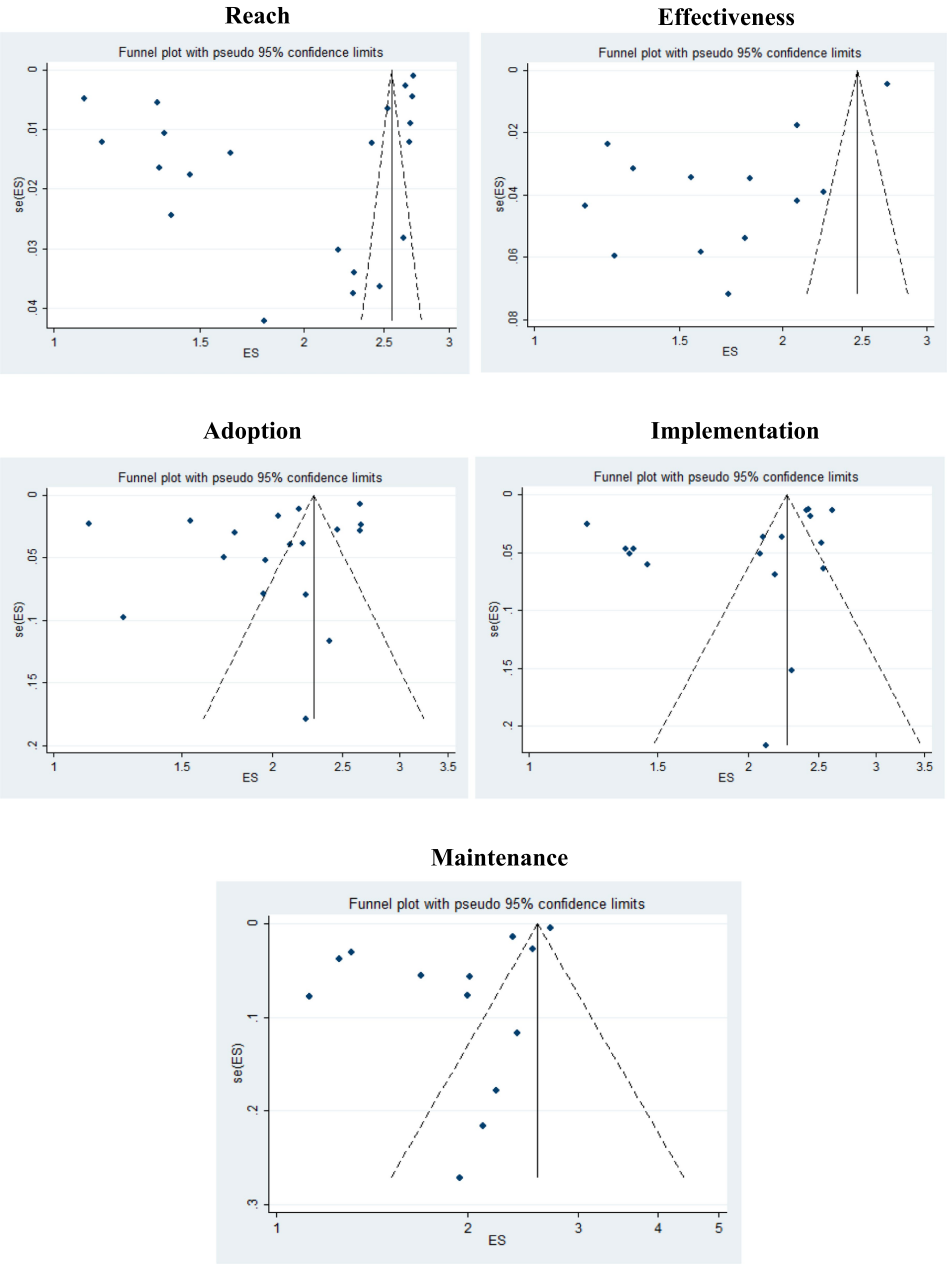
Funnel plot of the prevalence of RE-AIM dimensions, with 95% confidence limits. *ES: prevalence estimates grouped from the studies; se(ES): Standard error of the estimate.

Figure 3 shows the funnel graphs for visualizing the risk of publication bias. In these analyses, it is possible to observe asymmetry between the investigations. This asymmetry is confirmed by Egger’s test, which showed significant results for “reach” (*p* = 0.023), “effectiveness” (*p* = 0.001) and “maintenance” (*p* = 0.016).

**Figure 4:**
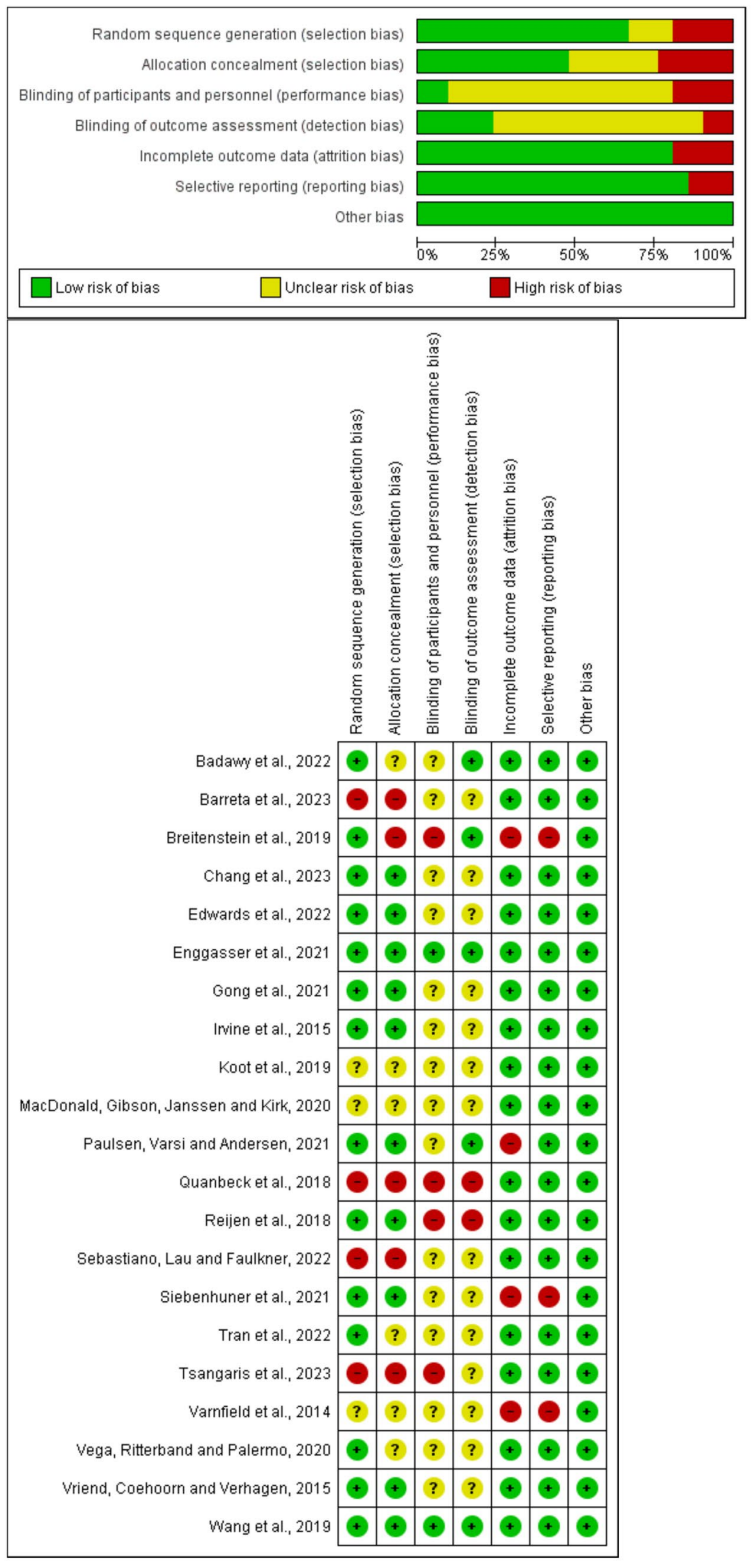
Summary risk of bias for each study included in the meta-analysis.

The risk of bias in each study was also analyzed (Figure 4). In summary, one article fully met the quality criteria, and 21 partially met these criteria. The majority of the articles (17/21) provided results and follow-up data, indicating a low risk of attrition bias. Concerning reporting bias, the majority (18/21) had a low risk of bias, as they clearly expressed the characteristics of the participants in their respective studies.

## Discussion

The results of this review reflect how the RE-AIM dimensions were reported in the included studies. More than 60% of the target population was reached, as described by the original authors. Adoption and implementation were the most frequently reported dimensions, with around 70% of individuals engaged according to each study’s criteria. Effectiveness was reported for 52% of participants, based on study-specific outcome definitions. These percentages refer these the reporting adequacy and presence of each RE-AIM dimension within the included studies, and do not directly correspond to clinical health outcomes or intervention success rates.

The “Reach” dimension refers to the level of individual participation, such as patients or health professionals, and involves the proportion and risk characteristics of people who are impacted by or participate in an intervention or program (13). The studies revealed that reach was 67% of the individuals evaluated, suggesting that most studies explicitly reported sample characteristics, as information such as advanced age, limited internet connection and low education, for example, can be a barrier to the reach of digital interventions. These results suggest the need for digital interventions that consider the diverse characteristics of individuals, seeking to serve mainly the most vulnerable, such as those with low income and less education, who have difficulty accessing services.

The “Effectiveness” dimension examines the impact of interventions, checking whether they have generated improvements or losses in the health and quality of life of those involved (13). In this review, effectiveness reached 52% of participants, indicating that not all interventions had a positive impact on those assessed. This can be explained by the lack of detail in some studies, which did not show a comparison of the results after the intervention or were unable to assess the improvements in patients’ health and quality of life after using the app. They also failed to compare those who completed the program with those who dropped out, to identify the real impact of the interventions. It is also important to think that the low effectiveness may be the result of low adherence to the app, as presented by the authors Chang et al. (26), who reported low acceptance of the intervention due to the stigmas associated with HIV.

The lower scores observed in the effectiveness dimension, compared to adoption or reach, may be justified because, while several strategies can be applied to increase the reach and adoption of interventions, health improvements require sustained behavioral changes, and monitoring and measuring these improvements in the digital context is complex. Furthermore, the duration of interventions is limited in most studies, which makes it difficult to detect significant health outcomes.

“Adoption” refers to the rate of acceptance and the representativeness of the places, such as work environments, health institutions, or communities, that implement a program or policy (13). This dimension reached 70% of participants, showing that the majority of those assessed, whether patients or healthcare staff, accepted using the apps for digital interventions. Information such as the number of downloads of the application, the number of login accesses, registrations in intervention programs, responses to questionnaires and interviews about the interventions, time spent using the application, and discontinuation of use, were presented in the evaluation of this dimension. Low adoption rates have been attributed to factors such as cost, insufficient training of professionals in the use of the digital tool, and the format and location of interventions (30, 31). According to Asare et al. (32), not disclosing team details, for example, does not allow us to know which resources need future interventions, which undermines both the effectiveness of the intervention and its adoption on an ongoing basis and in different scenarios.

Some important factors for increased adoption include personalized and timely support for users during implementation, the ability to adapt to patients’ routines, the inclusion of a two-way service model that allows communication between patients and healthcare professionals involved in care, and technological literacy, especially for older patients. Adoption tends to be higher when strategies are less intensive and supported by authorities in the field, in addition to media coverage that highlights the importance of such interventions (33).

The “Implementation” dimension assesses the degree to which an intervention or program is carried out as planned and with the necessary adaptations to achieve the proposed objectives (13). It impacted 68% of the participants in the studies, which highlighted the adaptations made and provided a detailed account of the team’s actions when assessing individuals both before and after the intervention. According to Stirman et al. (34), this information is important so that interventions can be adapted to different contexts while maintaining the essential characteristics. Furthermore, reporting the costs of implementing a given intervention allows future researchers to have a financial reference for developing similar studies (34).

The last dimension, “maintenance,” checks the extent to which interventions continue to be used in the long term, both by individuals and by organizations or communities, becoming part of their routines or practices (13). In the studies included, it reached 64% of participants, and it was reported that they continued to use the app even after the research or funding ended, or when contracts for its use were renewed. Satisfaction surveys and assessments of permanent behavior change were used to understand this dimension. Some studies also reported this dimension at the organizational level, which is important because it shows that the intervention has become part of the routine in health care.

Low retention rates have been attributed to researcher time and financial constraints, and to the lack of reports with generalized information on the impact of interventions (31, 32). According to Blackman et al. (30), it is essential to recognize that advances in new technologies can render current interventions obsolete. Furthermore, technical issues can result in decreased motivation to use the tools or lead to withdrawal from participation over time.

Research indicates that mobile applications play a significant role in healthcare by supporting monitoring, promoting treatment adherence, and enhancing health outcomes. Key features of these applications—such as adaptability to individual needs, the inclusion of health guidance through text and video, and functionality in offline settings—are identified as critical factors driving their utilization in health-related contexts. The studies by Quinn et al. (35) and Kim et al. (36) support these findings, as the authors suggest that incorporating a health education platform within the mobile app significantly enhances outcomes such as blood pressure, blood glucose, and lipid levels. Additionally, it promotes the development of self-care and self-monitoring of risk factors over time, leading to overall improvements in health and quality of life.

Of the 21 studies included (24–29, 39–42, 44–54), 13 reported interventions in primary care, highlighting the significance of digital interventions in preventing the onset of risk factors and the progression of disease. This evidence was also reported in another study (37), indicating that the mobile application inserted at the primary care level allows care, monitoring, and management of health conditions, facilitating patient adherence to treatments and optimizing the work of the health team.

It is important to mention the existence of other models for evaluating digital health interventions, such as the Mobile App Rating Scale (MARS) and the Consolidated Framework for Implementation Research (CFIR) (38). However, RE-AIM was defined for this review because it comprehensively addresses internal and external validity, allows for assessment at the organizational and individual level, and considers short- and long-term results. Furthermore, it allows for consideration of the different contexts and diverse populations that can benefit from mHealth interventions, with emphasis on the field of public health (43).

This study has limitations. First, the high heterogeneity of the included studies. In this regard, we performed subgroup analyses considering the year of publication, duration of the intervention, and location of the study, but we did not find statistically significant differences. It was not possible to analyze factors such as age group and sex, since most studies did not provide this information.

Second, the possible classification bias, since some inconsistencies in the operationalization and interpretation of the RE-AIM dimensions were identified in the studies, such as the variation in the measurement tools and the thresholds for each dimension, which limited the comparability of the results. Despite this, data extraction and categorization were conducted by two reviewers, by consensus, or by the judgment of a third reviewer.

Third, it was not possible to perform a sensitivity analysis of the estimates found, due to the heterogeneity of the studies. Fourth, there was evidence of publication bias for three of the five RE-AIM dimensions (“Reach,” “Effectiveness” and “Maintenance”), as indicated by the visual asymmetry in the funnel plots and the statistically significant results in the Egger test. This suggests that the observed effect sizes may be overestimated due to the selective reporting of positive findings in the published literature. Finally, we understand the possibility of underestimation or incorrect classification of the RE-AIM dimensions, as they were extracted from the studies and each author may have failed to report important aspects of interventions that have been implemented. We also emphasize that we found a scarcity of records on RE-AIM in the context of mental health, which does not necessarily reflect the lack of mHealth interventions in this context.

We recommend that future studies prioritize the detailing and transparency of information on each dimension, so that more robust comparisons are possible, contributing to the advancement of mHealth interventions based on consistent evidence.

Strengths of this study are highlighted. First, the comprehensive search of six large databases, comprising studies from 11 countries over 12 years (2011–2023), which minimized selection bias and increased the representativeness of the evidence, with different population and health contexts.

Second, conducting the review according to the PRISMA guidelines ensured methodological rigor, and the use of the Joanna Briggs Institute (JBI) tool showed low to moderate risk of bias, especially in the reporting and attrition domains, confirming the methodological quality of this review.

Third, the use of a predefined protocol for extraction and categorization of RE-AIM data increased consistency and reduced classification bias, despite variability between studies. Furthermore, the multiple study designs included (cross-sectional studies, cohort studies, and randomized clinical trials) also broadened the scope of evidence.

Fourth, the meta-analysis allowed for the calculation of pooled estimates for each dimension, providing a clearer synthesis of each dimension. Subgroup analyses and assessment of bias using funnel plots and Egger’s test confirm the transparency and methodological rigor of this review.

Finally, by focusing on a wide range of health outcomes and intervention contexts, multiple criteria related to research and important decisions for quality of life in the context of public healththis review contributes valuable insights to researchers, policymakers, and public health practitioners interested in the implementation and impact of digital health interventions guided by the RE-AIM framework.

## Conclusion

This study synthesized the evidence on mobile health app-based interventions using the RE-AIM framework to assess impact across five key dimensions: reach, effectiveness, adoption, implementation, and maintenance. The results show that these dimensions are useful for assessing how digital interventions were designed, delivered, and reported. In this review, interventions had broad population reach, were well accepted by individuals and organizations, and demonstrated robust implementation processes. However, effectiveness and maintenance were the most challenging dimensions, reflecting the need to improve long-term impact and ensure sustained use of these tools.

High adoption rates indicate that digital interventions have substantial potential for integration into clinical practice and individuals’ daily routines, especially when delivered through accessible and personalized platforms. However, the observed variation in effectiveness highlights the need to develop more targeted strategies that address not only app use but also patients’ specific health needs and conditions.

This review makes a significant contribution to public health by providing a comprehensive overview of how digital health interventions have been implemented and evaluated in a variety of healthcare settings. By highlighting strengths and areas for improvement, the study provides important insights for the future development of mobile health applications that can achieve better clinical and health promotion outcomes.

## Data Availability

The original contributions presented in the study are included in the article/Supplementary material, further inquiries can be directed to the corresponding author.
