# Supplementary material for: Evaluation of mobile health applications using the RE-AIM model: systematic review and meta-analysis
Source: Front Public Health. 2025 Aug 4;13:1611789. doi: 10.3389/fpubh.2025.1611789 (PMC12358350; doi:10.3389/fpubh.2025.1611789)
Supplement: Supplementary file 1 [file Data_Sheet_1.docx]

**Supplementary file**

Appendix I – Search strategy according to database.

| **(“RE-AIM” AND (“mobile phone” OR “digital health” OR telehealth))** | | | | | |
| --- | --- | --- | --- | --- | --- |
| **MEDLINE** | **SCOPUS** | **BVS** | **WEB OF SCIENCE** | **EMBASE** | **CINAHLS** |
| 154 | 116 | 605 | 103 | 177 | 146 |
| **(“RE-AIM” AND (“mobile phone” OR “digital health” OR telehealth) AND “intervention”)** | | | | | |
| **MEDLINE** | **SCOPUS** | **BVS** | **WEB OF SCIENCE** | **EMBASE** | **CINAHLS** |
| 96 | 82 | 211 | 66 | 78 | 89 |
| **(“RE-AIM” AND (“mobile phone” OR “digital health” OR telehealth) AND (“health program” OR “health intervention”))** | | | | | |
| **MEDLINE** | **SCOPUS** | **BVS** | **WEB OF SCIENCE** | **EMBASE** | **CINAHLS** |
| 17 | 21 | 138 | 11 | 15 | 15 |

Appendix II - Characterization of the studies included in the systematic review and meta-analysis

| N | Title  Year of publication | Authors,  Country,  Year of study | Study design | Name of mobile app | Sample size Study duration | Sample characterization | Key intervention | Key mobile app features | Data of the mobile app and Outcomes | Main conclusions | JBI (%) |
| --- | --- | --- | --- | --- | --- | --- | --- | --- | --- | --- | --- |
| 1 | Impact of the COVID-19 Pandemic on the Implementation of Mobile Health to Improve the Uptake of Hydroxyurea in Patients With Sickle Cell Disease: Mixed Methods Study  2022 | Sherif M Badawy et al.  USA.  2019 - 2020 | Closed, non-randomized cohort trial | InCharge Health | 64 patients and 42 providers (doctors, nurses and medical assistants)    24 weeks | Participants diagnosed with sickle cell anemia between the ages of 15 and 45, treated with hydroxyurea. | Improving adherence to treatment with hydroxyurea in patients with sickle cell disease, and improving the correct prescription of hydroxyurea for the healthcare team. This multi-level strategy addresses determinants such as motivation, knowledge, self-efficacy and social support among patients, and knowledge, attitude and self-efficacy among providers | The app allows patients to record and monitor their adherence to treatment, sends reminders and notifications to patients about when to take their medication, provides educational information about hydroxyurea, including its benefits, side effects and the importance of adherence to treatment. | Use of the InCharge Health app varied among patients. We also found different barriers to implementing the app, especially those related to access to the healthcare system, with fewer face-to-face visits to the clinic and low contact between patients and providers, potentially reducing adherence to hydroxyurea. | mHealth apps are promising tools for improving adherence to hydroxyurea among adolescents and adults with sickle cell anemia. It allows them to manage their health conditions more effectively, promoting self-confidence and self-efficacy in adhering to treatment. | 90,9% |
| 2 | Public implementation of a web-based program for veterans with risky alcohol use and PTSD: A RE-AIM evaluation of VetChange  2021 | Justin L Enggasser, Nicholas A Livingston, Victoria Ameral, Deborah J Brief, Amy Rubin, Eric Helmuth, Monica Roy, Marika Solhan, Scott Litwack, David Rosenbloom, Terence M Keane.  USA. 2015 – 2017 | Cohort | VetChange | 760 returning military veterans  6 months | Returning veterans who served during the military conflicts in Iraq (Operation Iraqi Freedom) and Afghanistan (Operation Enduring Freedom, and New Dawn) and were experiencing alcohol-related problems. | VetChange is designed to help veterans develop the skills needed to reduce alcohol consumption to a safer level (i.e. moderation or abstinence). It is publicly available at no cost to the user. | It offers assessment tools, a drinking diary and skills and motivation training sections. Veterans receive personalized feedback on their alcohol consumption and symptoms of post-traumatic stress disorder. It has strategies for motivation, cognitive-behavioral training and self-control, tailored specifically to alcohol-related problems. | VetChange successfully reached the intervention's target population. Participants reported improvements in alcohol consumption, post-traumatic stress disorder and overall quality of life. | The study emphasized the importance of maintaining the program, both in terms of associated costs and the need for long-term institutional support. Analysis of six-month outcomes showed that the intervention could become part of standard practice in organizations that serve veterans. | 72,7% |
| 3 | Promoting healthy lifestyle in Chinese college students: evaluation of a social media-based intervention applying the RE-AIM framework  2021 | Mengying Wang, Yijing Guo, Yu Zhang, Sasa Xie, ZhiYing Yu, Jun Luo, Danyu Zhang, Zhaoyan Ming, Xiuyang Li, Min Yang  China. 2019 | Randomized Controlled Clinical Trial | WeChat, as a Zhishi mini-program | 110 participants (87 in the intervention group and 23 in the control group)    21 days | Undergraduate students from Zhejiang University, with a median age of 18. They used the app for health education, diet and physical activity supervision for 21 days. | Improving healthy lifestyles among university students with a 21-day program. The intervention included educational components (such as classes or information materials) and practical activities (such as exercise or diet monitoring). | It allows users to send text messages, voice messages, photos and videos to the group. Helps participants monitor their level of physical activity, their food intake, calculate calories, and assess diet quality through pattern recognition and artificial intelligence. | The student recruitment rate was 79.1%. The intervention group showed significant progress in terms of healthy food intake (all p < 0.05) and an improvement in blood pressure level (p = 0.004) over 21 days. Around 60.9% of the subjects were satisfied with the whole program and 64.4% would like to take part in the program again. | This intervention has shown a great improvement in healthy behavior with great viability for further dissemination. | 53,8% |
| 4 | Implementation findings from an effectiveness-implementation trial of tablet-based parent training in pediatric primary care  2020 | Susan M Breitenstein, Stacy Laurent, Laura Pabalan, Heather J Risser, Pamela Roper, Mary T Saba, Michael Schoeny.  USA. 2016 - 2018 | Randomized clinical trial | ez Parent | 759 parents    10 months | Parents of children ages 2 to 5 during a child care visit at four urban pediatric primary care sites in predominantly low-income communities in Chicago. | A training program for parents, designed for use in pediatric primary care settings. The intervention was introduced during childcare appointments for children aged two to five, allowing health providers to integrate the study presentation into their usual workflow. | Composed of six modules focused on the development of effective parenting skills and the use of behavioral strategies such as routines and praise. It has videos, activities, quizzes and tasks that help parents apply the strategies learned in a practical and engaging way, and can provide feedback on their experience. It is adapted to be culturally and contextually relevant, making it easier for specific groups to access. | 85% of participants completed all the program modules, 88% of users reported that the program was very useful, and 82% said they would recommend the intervention to other parents. During implementation, 78% of health providers implemented the program at least once, with positive comments. Barriers were identified, such as the lack of consistent information and insufficient time to effectively implement the program's activities. | The program generated modest improvements in parenting behaviors and children's outcomes, suggesting that it may be beneficial in promoting positive parenting practices and reducing the use of physical punishment. | 61,5% |
| 5 | Users' Perspectives, Opportunities, and Barriers of the Strengthen Your Ankle App for Evidence-Based Ankle Sprain Prevention: Mixed-Methods Process Evaluation for a Randomized Controlled Trial    2018 | Miriam van Reijen, Marianne Asscheman, Ingrid Vriend, Willem van Mechelen, Evert Verhagen    Holanda. 2017 | Randomized controlled clinical trial | Strengthen Your Ankle | 220 participants  8 weeks | Active participants (athletes), between 18 and 70 years of age, who had suffered an ankle sprain in the last two months. Participants were randomly assigned to the app or booklet intervention group and were instructed to follow the “Strengthen Your Ankle” neuromuscular training prevention program | Neuromuscular training program designed to prevent ankle sprains, especially in athletes who have already suffered this type of injury. | The app provides step-by-step instructions for carrying out the exercises, ensuring that users can perform them correctly (guided exercises), and monitors progress, which can help increase motivation and adherence. It allows feedback and reminders to be sent to users, helping to maintain regularity of practice. It offers information on injury prevention and the importance of neuromuscular training. | Adherence to the program increased over time, from 23% to 45% in randomized controlled trials. There were no significant differences in lesion density between users of the app and those who used a printed booklet. Both methods were comparable in terms of cost-effectiveness. App users reported that the convenience of having the app on their cell phone, the visual support and the reminder functions were positive aspects. | The app proved to be effective in reducing the incidence of ankle sprains for those who showed high adherence to the training. Despite the program's proven effectiveness, adherence remains a challenge. Although the number of downloads of the app is significant, it represents a small fraction of the potential target audience, indicating that there is still great scope for increasing the reach and adoption of the program | 69,2% |
| 6 | Mobile-Web app to self-manage low back pain: randomized controlled trial    2015 | A Blair Irvine, Holly Russell, Michael Manocchia, David E Mino, Terri Cox Glassen, Rebecca Morgan, Jeff M Gau, Amelia J Birney, Dennis V Ary   USA. 2015 | 3-arm randomized controlled trial | FitBack | 597 participants: (1) treatment group (n=199), who used the FitBack intervention, (2) alternative care group (n=199), who received 8 emails with links to 6 websites with information on low back pain, or (3) usual care control group (n=199), who only received emails as prompts to complete the assessments.  4 months | Participants recruited through collaborating companies had to (1) be between 18 and 65 years old and live in the United States | Provide education and behavioral strategies to help adults manage non-specific low back pain and prevent future episodes of pain. Uses a self-adjusted cognitive-behavioral approach, allowing users to choose the strategies that best suit their individual needs. | The app has a tool that allows users to track their daily pain management activities, categorized into four areas: rest and relief, mindfulness, general fitness and specific back pain exercises. Simple 7-day and 30-day Trend Charts to help users identify trends in pain levels in relation to self-help activities. Short educational videos (1-4 minutes) covering general aspects of pain and management strategies, as well as instructional videos on strengthening and stretching exercises adapted to different types of work. Weekly email messages on self-care, pain and reminders to return to the FitBack program. | FitBack users showed a significant reduction in low back pain compared to the control and alternative care groups. They showed improvements in critical physical, behavioral and workplace outcome measures at a 4-month follow-up. This included improved functionality, quality of life and well-being compared to the control group. The majority of participants (96-98%) reported understanding the program's recommendations. | The study suggests that app-based interventions like FitBack could be a cost-effective solution for reaching many people. FitBack's self-adjusting approach, which allows users to choose strategies that suit their individual needs, was a key factor in the program's success. It highlights FitBack's potential as a valuable tool for managing low back pain, promoting self-management and patients' responsibility for their own health. | 76,9% |
| 7 | REDCap mobile data collection: Using implementation science to explore the potential and pitfalls of a digital health tool in routine voluntary medical male circumcision outreach settings in Zimbabwe    2022 | Vi Tran, Farai Gwenzi, Phiona Marongwe, Olbarn Rutsito, Pesanai Chatikobo, Vernon Murenje, Joseph Hove, Tinashe Munyaradzi, Zoe Rogers, Mufuta Tshimanga, Vuyelwa Sidile-Chitimbire, Sinokuthemba Xaba, Gertrude Ncube, Lewis Masimba, Batsirai Makunike-Chikwinya, Marrianne Holec, Scott Barnhart, Bryan Weiner, Caryl Feldacker    Zimbábue. 2019 – 2020 | Cross-sectional | REDCap Mobile | 35 nurses (17 from Partner 1 and 18 from Partner 2)    9 months | Nurses who worked in male circumcision programs in rural health settings. These nurses were associated with two partnerships (Partner 1 and Partner 2) that implemented REDCap Mobile for data collection. | Implementation of the REDCap Mobile application for data collection in male circumcision programs in health settings in Zimbabwe, replacing paper forms. It allowed data to be collected in real time, with the ability to synchronize information when an internet connection was available. | Allows users to collect data even without an internet connection. Offers the ability to create customized forms for data collection, adapting to the specific needs of health programs. Allows monitoring and evaluation teams to review the data collected, making it easier to identify areas that need additional supervision or improvement. Allows health teams to work simultaneously. | After implementation, there was 100% adoption of REDCap Mobile by the team, resulting in the discontinuation of the use of paper reports by October 2019. The intention to maintain the use of REDCap Mobile was evidenced by programmatic activities, such as conducting informal interviews with users and collecting feedback on the application. Challenges: need for ongoing training, connectivity problems, and the importance of ensuring that all users update the app for optimization. | It has shown potential for improving the quality of data collected in health programs, especially in contexts of low connectivity, with improvements in data completeness in some teams, helping to identify areas for improvement and adjustments to the application. | 87,5% |
| 8 | Feasibility of implementing patient-reported outcome measures into routine breast cancer care delivery using a novel collection and reporting platform  2023 | Elena Tsangaris, Colby Hyland, George Liang, Joanna O'Gorman, Dany Thorpe Huerta, Ellen Kim, Maria Edelen e Andrea Pusic  USA. 2021-2022 | Cohort | imPROVE | 2,961 patients  8 months | The majority of patients undergoing breast cancer-related surgery were aged 50-59, were of non-Hispanic origin, had a university degree, were married or in a stable union, and more than half were undergoing reconstruction surgery. | A health information technology platform that enables the quantification of quality of life and the care experience from the patient's perspective. | Motivational messages, access to scheduled and on-demand assessments, direct links to resources by type and stage of treatment, library of educational materials, links to women's communities, personal notebook (things I want to ask the doctor, things I'm grateful for and reflections on my journey). Doctors have access to a list of patients, a summary of their medical history and categorized individual data. | Adherence to using the app was influenced by factors such as patient age, marital status and educational level. Older, unmarried and less educated patients had lower download and adherence rates. | The implementation of the app proved to be effective in engaging patients in the process of collecting data about their health during breast cancer treatment. Continuous feedback from clinical staff and patients was key to identifying and resolving barriers to adherence, such as technical problems and difficulties in use. They highlight the importance of a collaborative, patient-centered approach to implementing health technologies | 90,0% |
| 9 | A Mobile Lifestyle Management Program (GlycoLeap) for People With Type 2 Diabetes: Single-Arm Feasibility Study  2019 | David Koot, Paul Soo Chye Goh, Robyn Su May Lim, Yubing Tian, Teng Yan Yau, Ngiap Chuan Tan and Eric Andrew Finkelstein.  Singapura. 2017 | Cohort | GlycoLeap | 785 participants  24 weeks | Participants diagnosed with type 2 diabetes and with a glycated hemoglobin (HbA1c) of ≥7.5% in the two months prior to inclusion in the study. The average age of the participants was 53.5 years, they had a BMI of over 23 kg/m² and were not on insulin treatment. | Implementation of a lifestyle management program, which consisted of two main steps: online educational links about diabetes and healthy behaviors and the Glyco mobile app, which allowed for the recording and monitoring of blood glucose levels, weight, meals and physical activity. The app also included a health coaching feature, where accredited nutritionists provided personalized feedback and suggested opportunities for improvement. | Diabetes education classes, glucose and weight monitoring (manually entered by the patient), meal logging, physical activity tracking and an online fitness coach for questions and activity suggestions. | The participants showed a significant improvement in their HbA1c levels over the course of the study. Almost 20% of participants lost more than 5% of their initial weight. The study observed a high level of engagement in the first few weeks, but a decrease in use of the app over time. The app received good ratings in terms of usability and user satisfaction, suggesting potential for the sustainability of GlycoLeap as a long-term intervention. | GlycoLeap was shown to be effective in improving glycated hemoglobin levels and promoting weight loss among participants with type 2 diabetes mellitus, making it effective for diabetes management, while emphasizing the importance of strategies to improve user engagement and adherence over time. | 54,0% |
| 10 | Mixed Methods, Implementation Science Evaluation of a Community Health Worker Strategy for HIV Service Engagement in Uganda  2023 | Larry W. Chang, Rose Pollard, Ismail Mbabali, Aggrey Anok, Heidi Hutton, K. Rivet Amico, Xiangrong Kong, Jeremiah Mulamba, Joseph Ssekasanvu, Amanda Long, Alvin G. Thomas, Kristin Thomas, Eva Bugos, Kimiko van Wickle, Caitlin E. Kennedy, Gertrude Nakigozi.  Uganda. 2015 – 2018 | Community randomized clinical trial | Smartphone app | 1,903 participants  3 years and 3 months | Individuals aged between 15 and 49 who lived in the fishing community of Rakai. A total of 1,891 participants, of whom 580 reported having received counseling from community health workers, while 1,311 were not exposed to the intervention. The average age was 32.7 years, 35.8% were over 35, 34.8% were women and 32.5% were married. | Implementation of the community health worker program, which consisted of HIV counseling and promoting adherence to antiretroviral treatment. Use of the app to guide the agents in their visits and in structuring questions, supervision, and increasing access to health services, improving the acceptance and effectiveness of HIV treatment, especially in groups that traditionally had less access to these services. | The application allowed data to be recorded in real time, making it easier to monitor visits and interactions with customers. Agents were able to access protocols and scripts programmed into the app, which helped them conduct sessions in a more structured and effective way. It made it possible to monitor clients' progress in relation to treatment and adherence to health recommendations, allowing adjustments to be made to approaches as necessary. | There was a significant increase in HIV care coverage and adherence to antiretroviral treatment over time. The agents visited clients an average of four times during the study period. Acceptance of the agents' strategy was considered high among residents, they were seen as reliable resources, and the intervention was implemented in a manner consistent with the original conceptual model. In addition to increasing engagement in health services and helping to combat the stigma associated with HIV. | The strategy was widely accepted and resulted in an increase in HIV care coverage and adherence to antiretroviral therapy. Despite acceptance, adoption has been hampered by factors such as lifestyle and stigmas associated with HIV. | 73,0% |
| 11 | A RE-AIM Analysis of a Mental Health App for Undergraduate and Medical Students during the COVID-19 Pandemic: A Retrospective Cross-Sectional Study  2023 | Berreta K, Nguyen C, Stoner AM, Ridgeway L, Wilson A, Fadel N, Biber D.  USA. 2022 | Cross-sectional | Sharpen | 1,052 university students (270 medical students and 782 students from other undergraduate courses)  23 months | Undergraduate college students recruited from five universities in the Appalachian region, The Sharpen app was adapted to include resources relevant to each campus. | The Sharpen app was developed to provide mental health support for university students. | The app offered modules on mental health, socio-emotional learning, mindfulness-based stress reduction and suicide prevention. It allowed asynchronous access, customized with logos and resources specific to each university, as well as welcome videos from advisors and students. | Medical students viewed significantly more pages and had a higher average number of unique page views compared to students from other degree courses. All the universities recruited took part in the study and continued to use the Sharpen app after the end of data collection, resulting in a 100% retention rate. | The results of this research provided evidence that there was some difference in the way medical students consume mental health content compared to students on other courses. It highlighted the importance of mental health interventions tailored to students' needs and the effectiveness of the Sharpen app as a tool to support mental health in university settings. | 75,0% |
| 12 | An Evaluation of a Commercialized mHealth Intervention to Promote Physical Activity in the Workplace  2022 | Katie M. Di Sebastiano, Erica Y. Lau, Lira Yun e Guy Faulkner   Canada. 2017 - 2020 | Quasi-experimental | UPnGO | 1,980 employees from 17 organizations  12 months | Seventeen companies took part, from various sectors: public administration, finance, management, technical and scientific services, agriculture, energy and telecommunications. The sample was made up predominantly of sedentary individuals. | A system for monitoring physical activity using personal tracking devices, which allowed participants to keep track of their steps and healthy habits. | It allowed users to record and monitor their daily physical activity, including step counts and other activity metrics. Provided periodic reports on their physical activity progress. It promoted opportunities for interaction between participants, offered educational resources on physical activity behavior change and included monthly themes and quarterly challenges that encouraged participants to engage in physical activity. | Of the total, 98% of participants accessed the system at the beginning; 47% within six months; and 30% within 12 months. Of the participants, 42% tracked steps at the beginning, 34% within six months, and 25% within 12 months; 39% tracked healthy habits at the beginning, 10% within six months, and 7% within 12 months. Only two of the 17 organizations renewed their contracts beyond the first year, representing 12% maintenance of the program. | Although the program reached a significant number of participants and was well-intentioned, there was a substantial drop in engagement and effectiveness over time. The average step count decreased, and the adoption and implementation of the program also showed significant declines. This suggests that the sustainability of digital health interventions in workplaces can be challenging. | 55,5% |
| 13 | Process evaluation of the implementation of a decision support system to prevent and treat disease-related malnutrition in a hospital setting  2021 | Mari Mohn Paulsen, Cecilie Varsi e Lene Frost Andersen  Noruega. 2018-2020 | Randomized clinical trial | MyFood | 100 patients (49 in the intervention group and 51 in the control group)  120 nurses  During hospitalization | The study was carried out in the hematology department of a large university hospital in Norway. The patients in the intervention group were on average 50 years old and 71% were men. | Designed to help patients monitor and manage their nutritional intake during their hospital stay. The nurses acted as facilitators and supporters in the implementation of MyFood during the intervention. | The app allowed users to record the foods and drinks they ate, provided an assessment of their nutritional intake, generated reports that were sent to nurses, including personalized recommendations for nutritional support for patients, as well as receiving feedback on their caloric and nutritional intake. | Of all the patients, 88% reported that they became more aware of their nutritional situation after using MyFood. Of the nurses, 81% felt that using MyFood for dietary recording was better than traditional paper methods, 79% found it useful for monitoring patients at risk of malnutrition, and 72% believed that the app should be implemented in the Norwegian healthcare system. | MyFood empowered patients in relation to their nutritional situation, helping them to better understand how different types of food contributed to their nutritional needs and motivated them to eat more to achieve their nutritional goals, especially at times of low appetite. Adherence among patients was higher than among nurses, so overcoming barriers to adherence among healthcare professionals is crucial to maximizing their impact on nutritional care. | 76,9% |
| 14 | Implementing a Mobile Health System to Integrate the Treatment of Addiction Into Primary Care: A Hybrid Implementation-Effectiveness Study  2018 | Andrew Quanbeck, David H Gustafson, Lisa A Marsch, Ming-Yuan Chih, Rachel Kornfield, Fiona McTavish, Roberta Johnson, Randall T Brown, Marie-Louise Mares and Dhavan V Shah  USA. 2012 | Quasi-experimental | Seva | 268 patients  12 months | Patients with drug addiction seen in primary care. The age range varied from 21 to 64 years and 67.9% of the patients were white, | Designed to support patients with substance use disorders in managing their recovery. | The app allows users to interact and support each other, offering teaching on problem-solving skills, self-regulation and other competencies relevant to recovery. It includes relaxation exercises and cognitive-behavioral therapy strategies for dealing with cravings and high-risk situations. | Patients showed significant improvements, including: a 44% reduction in drinking days, a 34% reduction in illicit drug use days, improved quality of life and HIV screening rates, and a reduction in the number of hospitalizations. There was a high adoption rate, with 60% of patients accessing the app in the last week of the implementation period. | The data underscores Seva's effectiveness as a support tool in the recovery of patients with substance use problems, while also highlighting the need for strategies to improve the adoption and sustainability of the app in clinical settings. | 77,7% |
| 15 | Implementation of an app-based neuromuscular training programme to prevent ankle sprains: a process evaluation using the RE-AIM Framework  2014 | Ingrid Vriend; Iris Coehoorn; Evert Verhagen   Holanda. 2011 - 2013 | Cross-sectional | "Versterk je Enkel " | 82 participants  8 weeks | Athletes aged between 12 and 70 who had suffered a lateral ankle sprain up to two months prior to inclusion in the study. Recruited from medical (hospital emergency rooms, general clinics, physiotherapy offices) and medical channels (newspapers, magazines and sports tournaments, Internet). | Designed to prevent ankle sprains in athletes. | The app offers a series of exercises in videos demonstrating execution, feedback via messages and information on injury prevention. | The sample included users who varied in their frequency of use of the app: 38% (n=31 out of 82) did not actively use the app, 33% (n=27 out of 82) used the app frequently (several times a week). Others reported infrequent use. | The study indicated that the marketing strategy used may not have been the most effective for implementing the app. A better understanding of the target population and a more structured approach are recommended for future implementations of evidence-based prevention apps. | 62,5% |
| 16 | Telehealth for chronic disease management: do we need to RE-AIM?  2014 | Marlien Varnfield, Mohan Karunanithi, Hang Ding, Dominique Bird and Brian Oldenburg  Austrália. 2014 | Randomized controlled clinical trial | "Care Assessment Platform" | 120 people: 60 in traditional cardiac rehabilitation and 60 in the care assessment platform  6 weeks. | Post-myocardial infarction patients who were able to take part in cardiac rehabilitation, with an average age of 55 years, and a majority of males. | Smartphone-based cardiac rehabilitation. This model is designed to be an alternative to traditional cardiac rehabilitation, which is usually carried out in health centers. | The app allowed patients to record and monitor their physical activity, vital signs, access educational materials on heart health, including information on exercise, nutrition and stress management. Patients could participate in weekly consultations with health professionals, who offered support and motivation, as well as answering questions and providing personalized guidance. | The group that used the app had higher adherence and treatment completion rates compared to the group that didn't use it. They reported greater satisfaction with the flexibility and support offered by the program compared to the traditional model. | There was potential to improve self-management of chronic conditions, but adoption and implementation in traditional health systems still face challenges. More studies are needed to assess the maintenance of behaviors and the integration of interventions into existing services. | 76,9% |
| 17 | Impact of community health worker intervention on PrEP knowledge and use in Rakai, Uganda: A mixed methods, implementation science evaluation  2022 | Edwards AJ, Pollard R, Kennedy CE, Mulamba J, Mbabali I, Anok A, Kong X, Nakyanjo N, Ddaaki W, Nishimura H, Wawer M, Amico KR, Hutton HE, Nakigozi G, Chang LW  Rakai, Uganda. 2015-2018 | Cohort | Smartphonand app | 1848 people who received the intervention from community health workers.  3 years and 3 months | They are mainly made up of fishermen, who are considered to be at high risk of HIV infection, aged between 15 and 49, married and with varying levels of schooling. | Implementation of a program, led by community health workers, aimed at increasing knowledge and use of prophylaxis interventions in the community. | The app allowed for the collection of data on the implementation of the intervention, including information on the number of individuals who received counseling and their experiences. It provided reports on the progress of the intervention, and HIV education materials. | The data showed that 92.7% of the participants received the counseling module on the interventions, but their use was 13.8% for “ever” use and 7.1% for “current” use. Knowledge about the interventions increased significantly among the participants who used the app, and the information received from the agents was a positive factor in the acceptance of the interventions to protect against HIV. | The main conclusions indicate that the Health Scouts intervention had a positive impact on knowledge and acceptance of the interventions, although there were challenges in acceptance among some groups, such as men. The mixed approach allowed for a deeper understanding of the effectiveness of the intervention. | 90,9% |
| 18 | Improvements in Health Might Contradict Adherence to Mobile Health Interventions: Findings from a Self-Care Cancer App Study  2020 | Alexander Siebenhüner, Michael Mikolasek, Claudia M. Witt, Jürgen Barth.   Suíça. 2016-2017 | Cohort | CanRelax | 100 cancer patients  10 weeks | Oncology patients, 78.3% of whom were women, aged between 41 and 55, 43.4% diagnosed with breast cancer. | Mobile application that offered mindfulness and relaxation exercises, with audio instructions. It was designed to help users manage stress and improve their quality of life through self-care practices. | The app offered three types of exercises in audio format, which included: mindfulness meditation, guided imagination and progressive muscle relaxation. It reminded users to practice the exercises, which were also accessible offline, allowing patients to use them at any time. | Adherence to the app was high, with 65% of patients using the app until the end of the study. Patients who continued to use the app showed a decrease in stress levels and an improvement in quality of life over time. The analysis indicated that adherence to the app was associated with a reduction in stress, suggesting that continued practice could be motivated by the search for stress relief. | Although cancer patients can benefit from mobile health interventions, adherence to using the app can be challenging, especially in relation to improved health, which can contradict the motivation to continue using the app. | 63,6% |
| 19 | The Implementation of a Primary Care-Based Integrated Mobile Health Intervention for Stroke Management in Rural China: Mixed-Methods Process Evaluation  2021 | Enying Gong, Sun Li-xin, Qian Long, Hanzhang Xu, Wanbing Gu, Janet Prvu Bettger, Jingru Tan, Jixiang Ma, Tazeen H. Jafar, Brian Oldenburg, Lijing L. Yan  China. 2020 -2021 | Randomized controlled trial  controlled | SINEMA app | 637 patients  12 months | Patients diagnosed with stroke. Located in 25 rural Chinese villages who used local clinics as their first point of contact with the health system. Most were men, average age 65.7 years; 86.1% had ischemic stroke and 13.6% had hemorrhagic stroke. | A mobile health system aimed at improving stroke management by integrating primary care and mobile technology. | It included components aimed at both doctors and patients. Doctors received training and virtual group support. Patients received monthly follow-up visits and daily voice messages. | Of the participants, 90% received all follow-up visits according to the protocol and 50% received daily voice messages. Most of the intervention's components were adopted by the doctors, resulting in significant improvements in blood pressure control, medication adherence and the patients' quality of life. The intervention led to a reduction in disability, stroke recurrences and deaths among survivors. | The intervention showed potential for improving stroke management in rural areas, highlighting the importance of integrating mobile technology with primary care to meet the health needs of the rural population. | 84,6% |
| 20 | Assessing digital health implementation for a pediatric chronic pain intervention: Comparing the re-aim and bit frameworks against real-world trial data and recommendations for future studies  2021 | Rocio de la Vega; Lee Ritterband ; Tonya M Palermo  USA. 2020 | Randomized clinical trial | WebMAP Mobile (WMM) | 143 adolescents (73 in the intervention group and 70 in the usual care group)  8 months | Adolescents with chronic pain, present for at least three months, seen at  in eight clinics in the United States (pain clinics and  gastroenterology  specialized). | Digital psychological intervention program based on cognitive-behavioral therapy (CBT) for the self-management of chronic pain. | It includes modules on topics such as chronic pain education, recognizing stress and negative emotions, operant strategies, sleep hygiene, communication and relapse prevention. The aim is to help adolescents manage their pain and improve their quality of life. | Of the participants, 93% downloaded the app and used it after the first login, and 85.7% accepted the treatment; 40% of the participants completed the treatment and 88% of the clinics continued to recommend WMM after the end of the study. | WMM showed excellent reach and adoption, with good acceptance among users and providers. Efficacy was limited, but increased engagement in treatment was associated with reductions in pain and disability. Sustainability was evidenced by continued referrals by clinics after the study, and the app was made publicly available after the study ended. | 76,9% |
| 21 | A mixed methods evaluation of a digital intervention to improve sedentary behaviour across multiple workplace settings  2020 | Bradley MacDonald *, Ann-Marie Gibson, Xanne Janssen and Alison Kirk  Reino Unido. 2020 | Quasi-experimental | Welbot. | 137 participants  6 months | The participants had an average age of 34. The sample was  predominantly white Europeans, and participants reported sitting for 77.3% of the working day.  sitting for 77.3% of the working day. | The participants had an average age of 34. The sample was predominantly made up of white Europeans, and the participants reported sitting for 77.3% of the working day. | The app sent reminders and suggestions for users to get up and do physical activities, such as stretching or short walks, during the working day. It allows monitoring of sedentary behavior, including time spent sitting, standing and moving, provides reports on progress and includes educational resources. | The study showed a significant increase in standing time for the total group, with a 5% increase between baseline and the one-month follow-up. There was an increase in transitions per hour, indicating that the participants were getting up more frequently during the working day. | The study concluded that the digital intervention had a positive impact on reducing sedentary behavior and improving employee well-being, highlighting the importance of strategies adapted to different work contexts and the need for engagement at all levels of the organization to increase the adoption and effectiveness of the program. | 66,6% |

Appendix III – Manuscripts excluded and reason for exclusion.

| **Title of manuscript excluded** | **Reason for exclusion** |
| --- | --- |
| Changing dimensions in immunization: Implementation research on the E-tracker intervention for improving vaccination of children in Dhaka and Moulvibazar, Bangladesh | Pilot study |
| Dissemination and Implementation of a Google Apple Exposure Notification System for COVID-19 Risk Mitigation at a National Public University: Protocol for a Pilot Evaluation Study in a Real-World Setting | Pilot study |
| Integrating addiction treatment into primary care using mobile health technology: Protocol for an implementation research study | Study Protocol |
| Studies in Health Technology and Informatics | Not original article |
| A simple and sensitive ultra-high performance liquid chromatography tandem mass spectrometry method for the quantitative analysis of VX-548 in monkey plasma: Method validation and application to pharmacokinetic study. | Did not address the topic Did not use mHealth app |
| Implementation of a virtual ward as a response to the COVID-19 pandemic. | Did not address the topic Did not use mHealth app |
| Increasing the public health impact of evidence-based interventions in behavioral medicine: new approaches and future directions. | Did not use mHealth app Did not use RE-AIM |
| A Digital Health Intervention Platform (Active and Independent Management System) to Enhance the Rehabilitation Experience for Orthopedic Joint Replacement Patients: Usability Evaluation Study. | Did not use RE-AIM |
| Review on the impact of cell phone radiation effects on green plants. | Not original article |
| Tribally-led mobile outreach: improving access to harm reduction services in one rural reservation community. | Did not address the topic Did not use mHealth app |
| Breast Cancer Education and Empowerment in Ethiopia: Evaluating Community-Based Cancer Prevention Efforts Using the RE-AIM Framework. | Did not use mHealth app Did not use RE-AIM |
| A systematic search, heuristic evaluation and analysis of dental trauma mobile applications. | Not original article |
| Evaluation of the Effectiveness of a Bilingual Nutrition Education Program in Partnership with a Mobile Health Unit. | Did not use mHealth app Did not use RE-AIM |
| Acceptability of two mobile applications to support cross-sectoral, person-centred and empowering stroke rehabilitation - a process evaluation. | Did not use RE-AIM |
| Detrimental effects of electromagnetic radiation emitted from cell phone on embryo morphokinetics and blastocyst viability in mice. | Did not address the topic Did not use mHealth app |
| Outcomes and complications of Total Hip Arthroplasty in patients with a pre-existing pathologic spine-hip relation. A systematic review of the literature. | Not original article |
| E-health intervention for preventing recurrent ankle sprains: a randomised controlled trial in general practice. | Did not use RE-AIM |
| Eita! Reaching Communities and Young People to Drive Demand for Oral Pre-Exposure Prophylaxis in South Africa. | Did not use mHealth app Did not use RE-AIM |
| Applications of remote sensing vis-à-vis machine learning in air quality monitoring and modelling: a review. | Not original article |
| Insights from the scale-up and implementation of the Deadly Liver Mob program across nine sites in New South Wales, Australia, according to the RE-AIM framework. | Did not use mHealth app Did not use RE-AIM |
| A Complex mHealth Coaching Intervention to Prevent Overweight, Obesity, and Diabetes in High-Risk Women in Antenatal Care: Protocol for a Hybrid Type 2 Effectiveness-Implementation Study. | Study Protocol |
| Effectiveness of nicotine salt vapes, cytisine, and a combination of these products, for smoking cessation in New Zealand: protocol for a three-arm, pragmatic, community-based randomised controlled trial. | Did not use mHealth app Did not use RE-AIM |
| Athletes' experiences of using a self-directed psychological support, the BAck iN the Game (BANG) smartphone application, during rehabilitation for return to sports following anterior cruciate ligament reconstruction. | Did not use RE-AIM |
| Preclinical PK investigation of a novel IDO1/TDO dual inhibitor-SHR9146 in mouse plasma and tissues by LC-MS/MS. | Did not address the topic Did not use mHealth app |
| A personalized app to improve quality of life of patients with a stoma: A protocol for a multicentre randomized controlled trial. | Did not use RE-AIM |
| Long-term results of per-operative knee arthroscopy in confirming suitability for unicompartmental arthroplasty. | Did not use mHealth app Did not use RE-AIM |
| Revision of total knee arthroplasty with press-fit condylar SIGMA TC3-mobile bearing system and porous metaphyseal sleeves in type AORI type II and III bone defects. A long-term follow-up study. | Did not address the topic Did not use mHealth app |
| Total femoral arthroplasty for non-oncological indications. | Did not address the topic Did not use mHealth app |
| Realist review of community coalitions and outreach interventions to increase access to primary care for vulnerable populations: a realist review. | Not original article |
| Comparative pharmacokinetics of six bioactive components of Shen-Wu-Yi-Shen tablets in normal and chronic renal failure rats based on UPLC-TSQ-MS/MS. | Did not address the topic Did not use mHealth app |
| Efficacy of perturbation-based balance training in anterior cruciate ligament tears. A systematic review | Not original article |
| Self-Management Exercises Intervention on Text Neck Syndrome Among University Students Using Smartphones. | Did not use mHealth app Did not use RE-AIM |
| Improving prehospital traumatic shock care: implementation and clinical effectiveness of a pragmatic, quasi-experimental trial in a resource-constrained South African setting. | Did not use mHealth app Did not use RE-AIM |
| Fixed-bearing is superior to mobile-bearing in lateral unicompartmental knee replacement: a retrospective matched-pairs analysis. | Did not address the topic Did not use mHealth app |
| Protocol for the implementation of a statewide mobile addiction program. | Did not use mHealth app Did not use RE-AIM |
| A scoping review of mHealth technologies for opioid overdose prevention, detection and response. | Not original article |
| Community paramedic hospital reduction and mitigation program: study protocol for a randomized pragmatic clinical trial. | Did not use mHealth app Did not use RE-AIM |
| Similar Survivorship but Different Revision Reasons for Uncemented Mobile-Bearing and Cemented Fixed-Bearing Medial UKA: A Long-Term Population-Based Cohort Study of 2,015 Patients. | Did not address the topic Did not use mHealth app |
| The effects of transpositions of functional I retrotransposons depend on the conditions and dose of parental exposure. | Did not address the topic Did not use mHealth app |
| Midterm Outcomes in Lateral Unicompartment Knee Replacement: The Effect of Patient Age and Bearing Choice. | Did not address the topic Did not use mHealth app |
| The experiences and perceptions of wellbeing provision among English ambulance services staff: a multi-method qualitative study. | Did not address the topic Did not use mHealth app |
| Status quo and problem analysis of cervical cancer screening program in China: Based on RE-AIM framework. | Did not use mHealth app Did not use RE-AIM |
| Probing cellular health at the muscle level-Multi-frequency bioimpedance in Parkinson's disease. | Did not address the topic Did not use mHealth app |
| Introducing block-Toeplitz covariance matrices to remaster linear discriminant analysis for event-related potential brain-computer interfaces. | Did not address the topic Did not use mHealth app |
| The experience of staff utilizing data to evaluate and improve person-centred practice: An action research study. | Did not address the topic Did not use mHealth app |
| A Deep Learning-Based Facial Acne Classification System. | Did not address the topic Did not use mHealth app |
| The Response by International Emergency Medical Teams Following the Beirut Harbor Explosion in 2020 - Who Were They, When Did They Arrive, What Did They Do, and Were They Needed? | Did not address the topic Did not use mHealth app |
| Evaluation of the Implementation and Effectiveness of a Mobile Health Intervention to Improve Outcomes for People With HIV in the Washington, DC Cohort: Study Protocol for a Cluster Randomized Controlled Trial. | Study Protocol |
| [River-Lake States in the Tributary of the Three Gorges Reservoir Area and Their Effects on the Phosphorus Content of Different Forms in the Sediment]. | Did not address the topic Did not use mHealth app |
| High Reoperation Rate in Mobile-Bearing Total Ankle Arthroplasty in Young Patients. | Did not address the topic Did not use mHealth app |
| Prevention of Emotional Disorders and Symptoms Under Health Conditions: A Pilot Study using the Unified Protocol in a Fertility Unit | Did not use mHealth app Did not use RE-AIM |
| Effective engagement of a stakeholder advisory board in severe mental illness (SMI) research: A case study of a clinical trial to improve adherence among people with SMI and hypertension. | Did not use mHealth app Did not use RE-AIM |
| Remodulation of neurosurgical activities in an Italian region (Emilia-Romagna) under COVID-19 emergency: maintaining the standard of care during the crisis. | Did not address the topic Did not use mHealth app |
| Mid-Term Results After 517 Primary Total Hip Arthroplasties With a Shortened and Shoulderless Double-Taper Press-Fit Stem: High Rates of Aseptic Loosening. | Did not address the topic Did not use mHealth app |
| E-consensus on telemedicine in colorectal surgery: a RAND/UCLA-modified study. | Not original article |
| Intermediate to long-term clinical outcomes and survival analysis of the Salto Mobile Bearing total ankle prothesis. | Did not address the topic Did not use mHealth app |
| Lower revision rates for cemented fixation in a long-term survival analysis of three different LCS designs. | Did not address the topic Did not use mHealth app |
| Ginsenoside Contents in Ginseng: Quality by Design-Coupled Two-Dimensional Liquid Chromatography Technique. | Did not address the topic Did not use mHealth app |
| Revision Rates After Total Ankle Replacement: A Comparison of Clinical Studies and Arthroplasty Registers. | Not original article |
| Comparison of five-year clinical outcomes of 524 cemented and cementless medial unicompartmental knee replacements. | Did not address the topic Did not use mHealth app |
| A Clinical Risk Model for Surgical Site Infection Following Pediatric Spine Deformity Surgery. | Did not address the topic Did not use mHealth app |
| Regression Equations for Weight Estimation in Paediatric Resuscitation. | Did not address the topic Did not use mHealth app |
| Are the functional outcomes really inferior following unicondylar knee arthroplasty in patients with partial-thickness cartilage loss? | Did not address the topic Did not use mHealth app |
| Women's lived experiences of advanced cervical cancer: a descriptive qualitative study. | Did not address the topic Did not use mHealth app |
| Pattern of Repetitive Element Transcription Segregate Cell Lineages during the Embryogenesis of Sea Urchin Strongylocentrotus purpuratus. | Did not address the topic Did not use mHealth app |
| Demonstrating the processes and outcomes of a rural Community Mental Health Rehabilitation Service: A realist evaluation. | Did not address the topic Did not use mHealth app |
| Telemedicine follow-ups for COVID-19: Experience in a tertiary hospital. | Did not use mHealth app Did not use RE-AIM |
| Survival of Mycobacterium abscessus complex organisms on coins. | Did not address the topic Did not use mHealth app |
| User-centered requirements engineering to manage the fuzzy front-end of open innovation in e-health: A study on support systems for seniors' physical activity. | Did not use RE-AIM |
| Diagnostic Difficulties and Complexities in the Management of Dermatofibrosarcoma Protuberans in the Breast: A Case Report and Review of the Literature. | Not original article |
| Functional Cortical Connectivity Related to Postural Control in Patients Six Weeks After Anterior Cruciate Ligament Reconstruction. | Did not address the topic Did not use mHealth app |
| Risk of revision for medial unicompartmental knee arthroplasty according to fixation and bearing type : short- to mid-term results from the Dutch Arthroplasty Register. | Did not address the topic Did not use mHealth app |
| Development of an integrated model of care for allogeneic stem cell transplantation facilitated by eHealth-the SMILe study. | Did not address the topic Did not use mHealth app |
| Identifying barriers and facilitators to implementation of community-based tuberculosis active case finding with mobile X-ray units in Lima, Peru: a RE-AIM evaluation. | Did not use mHealth app Did not use RE-AIM |
| Same same-but different: using qualitative studies to inform concept elicitation for quality of life assessment in telemedical care: a request for an extended working model. | Did not address the topic Did not use mHealth app |
| [Determination of nine ginsenosides in health foods by solid extraction phase-ultra performance liquid chromatography-tandem mass spectrometry]. | Did not address the topic Did not use mHealth app |
| Is Pathfinder a safe alternative to the emergency department for older patients? An observational analysis. | Did not address the topic Did not use mHealth app |
| A validated UHPLC-MS/MS method for simultaneous determination of lumiracoxib and its hydroxylation and acyl glucuronidation metabolites in rat plasma: Application to a pharmacokinetic study. | Did not address the topic Did not use mHealth app |
| Sexual and reproductive health self-care interventions in the Eastern Mediterranean Region: findings from a cross-sectional values and preferences survey to inform WHO normative guidance on self-care interventions. | Did not use mHealth app Did not use RE-AIM |
| A Resilience-Building App to Support the Mental Health of Health Care Workers in the COVID-19 Era: Design Process, Distribution, and Evaluation. | Did not address the topic Did not use RE-AIM |
| E-consensus on telemedicine in proctology: A RAND/UCLA-modified study. | Did not address the topic Did not use mHealth app |
| The impact of splinting mobile mandibular incisors on Oral Health-Related Quality of Life-Preliminary observations from a randomized clinical trial. | Did not address the topic Did not use mHealth app |
| Eye-tracking glasses in face-to-face interactions: Manual versus automated assessment of areas-of-interest. | Did not address the topic Did not use mHealth app |
| Mobile App-Based Intervention for Pregnant Women With Stress Urinary Incontinence: Protocol for a Hybrid Effectiveness-Implementation Trial. | Study Protocol |
| Virtual visits for chronic neurologic disorders during COVID-19 pandemic. | Did not use mHealth app Did not use RE-AIM |
| Morphological and cytophysiological changes in selected lines of normal and cancer human cells under the influence of a radio-frequency electromagnetic field. | Did not address the topic Did not use mHealth app |
| Electromagnetic Waves from Mobile Phones may Affect Rat Brain During Development. | Did not address the topic Did not use mHealth app |
| Smartphone electrocardiogram for QT interval monitoring in Coronavirus Disease 2019 (COVID-19) patients treated with Hydroxychloroquine. | Did not use mHealth app Did not use RE-AIM |
| Nursing Teleconsultation for the Outpatient Management of Patients with Cardiovascular Disease during COVID-19 Pandemic. | Did not use mHealth app Did not use RE-AIM |
| You're only there on the phone'? A qualitative exploration of community, affect and agential capacity in HIV self-testing using a smartphone app. | Did not use RE-AIM |
| Healthcare stakeholders' perceptions and experiences of factors affecting the implementation of critical care telemedicine (CCT): qualitative evidence synthesis. | Not original article |
| Simultaneous Determination of Saponins and Lignans in Rat Plasma by UPLC- MS/MS and its Application to a Pharmacokinetic Study of Shenqi Jiangtang Granule. | Did not address the topic Did not use mHealth app |
| Impact of COVID-19 pandemic on the management of paediatric inflammatory bowel disease: An Italian multicentre study on behalf of the SIGENP IBD Group. | Did not use mHealth app Did not use RE-AIM |
| The use of an O-arm in endonasal endoscopic operations of the skull base. | Did not address the topic Did not use mHealth app |
| Attitudes of healthy volunteers to genetic testing in phase 1 clinical trials. | Did not address the topic Did not use mHealth app |
| Demystifying diabetes health coaching: A scoping review unveiling the 'who' and 'where' of health coaching for adults with type 2 diabetes. | Not original article |
| Type-2 diabetes primary prevention program implemented in routine primary care: a process evaluation study. | Not original article |
| Implementation and evaluation of a community-based treatment for late-life hoarding. | Did not address the topic Did not use mHealth app |
| Involvement of informal carers in discharge planning and transition between hospital and community mental health care: A systematic review. | Not original article |
| Developing and testing a new tool to foster wind energy sector industrial skills. | Did not address the topic Did not use mHealth app |
| Cementless unicompartmental knee replacement achieves better ten-year clinical outcomes than cemented: a systematic review. | Not original article |
| Long-term exposure to electromagnetic radiation from mobile phones can cause considerable changes in the balance of Bax/Bcl2 mRNA expression in the hippocampus of mice. | Did not address the topic Did not use mHealth app |
| The Cassandra retrotransposon landscape in sugar beet (Beta vulgaris) and related Amaranthaceae: recombination and re-shuffling lead to a high structural variability. | Did not address the topic Did not use mHealth app |
| Mental health recovery and physical health outcomes in psychotic illness: Longitudinal data from the Western Australian survey of high impact psychosis catchments. | Did not address the topic Did not use mHealth app |
| Impact of SARS-CoV-2 on Provided Healthcare. Evidence From the Emergency Phase in Italy. | Not original article |
| Communication With Patients Before an Operation: Their Preferences on Method of Communication. | Did not use mHealth app Did not use RE-AIM |
| Clinical Challenges and Considerations in Management of Chronic Pain Patients During a COVID-19 Pandemic. | Did not address the topic Did not use mHealth app |
| Management of aseptic failure of the mobile-bearing Oxford unicompartmental knee arthroplasty. | Did not address the topic Did not use mHealth app |
| Face Memorization Using AIM Model for Mobile Robot and Its Application to Name Calling Function. | Did not address the topic Did not use mHealth app |
| Effects of a Collective Family-Based Mobile Health Intervention Called "SMARTFAMILY" on Promoting Physical Activity and Healthy Eating: Protocol for a Randomized Controlled Trial. | Did not use RE-AIM |
| Telemedicina con telemonitorización en el seguimiento de pacientes con COVID-19 | Did not address the topic Did not use RE-AIM |
| Time to change the times? Time of recurrence of ventricular fibrillation during OHCA. | Did not address the topic Did not use mHealth app |
| Rhythmic neural activity is comodulated with short-term gait modifications during first-time use of a dummy prosthesis: a pilot study. | Did not address the topic Did not use mHealth app |
| Validity of Smartphone Heart Rate Variability Pre- and Post-Resistance Exercise. | Did not address the topic Did not use mHealth app |
| Does the short-term exposure to radiofrequency electromagnetic field originating from mobile phone affect auditory functions as measured by Acoustic Admittance and Evoked Otoacoustic Emission tests? | Did not address the topic Did not use mHealth app |
| You're not just a medical professional': Exploring paramedic experiences of overdose response within Vancouver's downtown eastside. | Did not address the topic Did not use mHealth app |
| Does personalised text messaging influence patients' caries risk? | Did not use mHealth app Did not use RE-AIM |
| Early administration of steroids in the ambulance setting: Protocol for a type I hybrid effectiveness-implementation trial with a stepped wedge design. | Did not use mHealth app Did not use RE-AIM |
| The Impact of the COVID-19 Pandemic on Genitourinary Cancer Care: Re-envisioning the Future. | Not original article |
| How I do it: 1st stage revision TKA. | Did not address the topic Did not use mHealth app |
| The Simple Cholestatic Complaints Score is a valid and quick patient-reported outcome measure in primary sclerosing cholangitis. | Did not address the topic Did not use mHealth app |
| Liquid chromatography/mass spectrometry analytical determination of gabapentin transformation products by heterogeneous photocatalysis and environmental evaluation. | Did not address the topic Did not use mHealth app |
| Telemedicine in ophthalmology in view of the emerging COVID-19 outbreak. | Not original article |
| Post-surgery and recovery experiences following one- and two-stage revision for prosthetic joint infection-A qualitative study of patients' experiences. | Did not address the topic Did not use mHealth app |
| Determination of Selected Phthalates in Some Commercial Cosmetic Products by HPLC-UV. | Did not address the topic Did not use mHealth app |
| Total Ankle Arthroplasty Survivorship: A Meta-analysis. | Not original article |
| Radiofrequency electromagnetic field affects heart rate variability in rabbits. | Did not address the topic Did not use mHealth app |
| The Effects of Gamification and Oral Self-Care on Oral Hygiene in Children: Systematic Search in App Stores and Evaluation of Apps. | Systematic review |
| Effects of mobile phone radiation on buccal mucosal cells: A systematic review. | Not original article |
| Are Virtual Fracture Clinics During the COVID-19 Pandemic a Potential Alternative for Delivering Fracture Care? A Systematic Review. | Not original article |
| Comparison of Intraocular Pressure Changes Due to Exposure to Mobile Phone Electromagnetics Radiations in Normal and Glaucoma Eye. | Did not address the topic Did not use mHealth app |
| Experience Story: How Do We Re-Implement What Has Been Implemented? | Did not address the topic Did not use mHealth app |
| SiTe iC: A telemonitoring system for heart failure patients. | Did not use RE-AIM |
| [Assessment of the clinical efficacy of telemonitoring and distant counseling in patients with uncontrolled hypertension]. | Did not use RE-AIM |
| Educação permanente no serviço de atendimento pré-hospitalar móvel de urgência: revisão integrativa | Not original article |
| Is revision total hip arthroplasty through the direct anterior approach feasible? | Did not address the topic Did not use mHealth app |
| Consumer Perceptions of Wearable Technology Devices: Retrospective Review and Analysis. | Not original article |
| The effectiveness of germicidal wipes and ultraviolet irradiation in reducing bacterial loads on electronic tablet devices used to obtain patient information in orthopaedic clinics: evaluation of tablet cleaning methods. | Did not address the topic Did not use mHealth app |
| Effect of electromagnetic radiation on redox status, acetylcholine esterase activity and cellular damage contributing to the diminution of the brain working memory in rats. | Did not address the topic Did not use mHealth app |
| A Comprehensive Evaluation of Lateral Unicompartmental Knee Arthroplasty Short to Mid-Term Survivorship, and the Effect of Patient and Implant Characteristics: An Analysis of Data From the Dutch Arthroplasty Register. | Did not address the topic Did not use mHealth app |
| Parental decisional satisfaction after hypospadias repair in the United Kingdom. | Did not address the topic Did not use mHealth app |
| Overview of Randomized Controlled Trials in Total Knee Arthroplasty (47,675 Patients): What Have We Learnt? | Did not address the topic Did not use mHealth app |
| Tele-transitions of care (TTOC): a 12-month, randomized controlled trial evaluating the use of Telehealth to achieve triple aim objectives. | Did not use mHealth app Did not use RE-AIM |
| Smartphones and tooth brushing: content analysis of the current available mobile health apps for motivation and training. | Did not use RE-AIM |
| Supporting the intimate relationship needs of service users with psychosis: what are the barriers and facilitators? | Did not address the topic Did not use mHealth app |
| Design of the user interface for "Stappy", a sensor-feedback system to facilitate walking in people after stroke: a user-centred approach. | Did not use mHealth app Did not use RE-AIM |
| High Failure Rates for Unicompartmental Knee Arthroplasty in Morbidly Obese Patients: A Two-Year Minimum Follow-Up Study. | Did not address the topic Did not use mHealth app |
| Syndesmotic Overload in 3-Component Total Ankle Replacement. | Did not address the topic Did not use mHealth app |
| Ambulance use, distance and outcomes in patients with suspected cardiovascular disease: a registry-based geographic information system study. | Did not address the topic Did not use mHealth app |
| Wireless Breast Localization Using Radio-frequency Identification Tags: The First Reported European Experience in Breast Cancer. | Did not address the topic Did not use mHealth app |
| Seguimiento telemático de COVID-19: experiencia de un hospital terciario | Did not address the topic Did not use mHealth app |
| Validation of a Novel Device for the Knee Monitoring of Orthopaedic Patients. | Did not use mHealth app Did not use RE-AIM |
| MOBIlity assessment with modern TEChnology in older patients' real-life by the General Practitioner: the MOBITEC-GP study protocol. | Did not address the topic Did not use mHealth app |
| Influence of reduction quality on functional outcome and quality of life in treatment of tibial plafond fractures: a retrospective cohort study. | Did not address the topic Did not use mHealth app |
| Revision Risk for Total Knee Arthroplasty Converted from Medial Unicompartmental Knee Arthroplasty: Comparison with Primary and Revision Arthroplasties, Based on Mid-Term Results from the Danish Knee Arthroplasty Registry. | Did not address the topic Did not use mHealth app |
| Inhaler technique errors in Romanian patients with asthma - a multicenter study. | Did not address the topic Did not use mHealth app |
| Melatonin Modulates NMDA-Receptor 2B/Calpain-1/ Caspase-12 Pathways in Rat Brain After Long Time Exposure to GSM Radiation. | Did not address the topic Did not use mHealth app |
| Effect of electromagnetic field exposure on the transcription of repetitive DNA elements in human cells. | Did not address the topic Did not use mHealth app |
| Impact of integrated district level mental health care on clinical and social outcomes of people with severe mental illness in rural Ethiopia: an intervention cohort study. | Did not address the topic Did not use mHealth app |
| Reduced Revision Risk for Dual-Mobility Cup in Total Hip Replacement Due to Hip Fracture: A Matched-Pair Analysis of 9,040 Cases from the Nordic Arthroplasty Register Association (NARA). | Did not address the topic Did not use mHealth app |
| Twenty-year survivorship of a cemented mobile bearing Total Knee Arthroplasty. | Did not address the topic Did not use mHealth app |
| Effects of radiofrequency electromagnetic field exposure on neuronal differentiation and mitochondrial function in SH-SY5Y cells. | Did not address the topic Did not use mHealth app |
| Alteration of testicular regulatory and functional molecules following long-time exposure to 900 MHz RFW emitted from BTS. | Did not address the topic Did not use mHealth app |
| SMART: Study protocol for a sequential multiple assignment randomized controlled trial to optimize weight loss management. | Did not use RE-AIM |
| Community-Based Implementation of a Paraprofessional-Delivered Cognitive Behavioral Therapy Program for Youth Involved with the Criminal Justice System. | Did not address the topic Did not use mHealth app |
| The possible global hazard of cell phone radiation on thyroid cells and hormones: a systematic review of evidences. | Not original article |
| "Putting people in charge of their own health and care?" Using meta-narrative review and the example of online sexual health services to re-think relationships between e-health and agency. | Not original article |
| Total hip arthroplasty versus hemiarthroplasty for independently mobile older adults with intracapsular hip fractures. | Did not address the topic Did not use mHealth app |
| Evaluation of a mobile C-arm cone-beam CT in interstitial high-dose-rate prostate brachytherapy treatment planning. | Did not address the topic Did not use mHealth app |
| Characterization of the stability and dynamics of Tn6330 in an Escherichia coli strain by nanopore long reads. | Did not address the topic Did not use mHealth app |
| Dosimetric evaluation of electron total skin irradiation using gafchromic film and thermoluminescent dosimetry. | Did not address the topic Did not use mHealth app |
| Long-term Follow-up Results of Buechel-Pappas Ankle Arthroplasty. | Did not address the topic Did not use mHealth app |
| Reliability and accuracy of smartphones for paediatric infectious disease consultations for children with rash in the paediatric emergency department. | Did not use RE-AIM |
| The effect of naringenin on the pharmacokinetics of ibrutinib in rat: A drug-drug interaction study. | Did not address the topic Did not use mHealth app |
| Effect of 900-, 1800-, and 2100-MHz radiofrequency radiation on DNA and oxidative stress in brain. | Did not address the topic Did not use mHealth app |
| Conversion of painful tibiotalocalcaneal arthrodesis to total ankle replacement using a 3-component mobile bearing prosthesis. | Did not address the topic Did not use mHealth app |
| Technology-based tools and services for people with dementia and carers: Mapping technology onto the dementia care pathway. | Did not use mHealth app Did not use RE-AIM |
| Efficiency in stroke management from acute care to rehabilitation: bedside versus telemedicine consultation. | Did not use mHealth app Did not use RE-AIM |
| The effect of 2.45 GHz non-ionizing radiation on the structure and ultrastructure of the testis in juvenile rats. | Did not address the topic Did not use mHealth app |
| Process Evaluation of the Diabetes Canada Guidelines Dissemination Strategy Using the Reach Effectiveness Adoption Implementation Maintenance (RE-AIM) Framework. | Did not use mHealth app Did not use RE-AIM |
| Seventeen to Twenty Years of Follow-Up of the Low Contact Stress Rotating-Platform Total Knee Arthroplasty With a Cementless Tibia in All Cases. | Did not address the topic Did not use mHealth app |
| Mobile Wireless Low-intensity Transcranial Ultrasound Stimulation System for Freely Behaving Small Animals. | Did not address the topic Did not use mHealth app |
| Vivências de cuidado em saúde de moradores de Serviços Residenciais Terapêuticos | Did not address the topic Did not use mHealth app |
| Oxidative and mutagenic effects of low intensity GSM 1800 MHz microwave radiation. | Did not address the topic Did not use mHealth app |
| An evaluation of the Fondo de Inclusión Social Energético program to promote access to liquefied petroleum gas in Peru. | Did not address the topic Did not use mHealth app |
| Estimation of blood and bone marrow doses of thyroid carcinoma patients treated with 131I through gamma spectrometry. | Did not address the topic Did not use mHealth app |
| A review on the current situation and challenges of colistin resistance in poultry production. | Not original article |
| Survival of the low contact stress rotating platform total knee replacement is influenced by age: 1058 implants with a minimum follow-up of 10â¯years. | Did not address the topic Did not use mHealth app |
| Home-based balance training using Wii Fit™: a pilot randomised controlled trial with mobile older stroke survivors. | Did not use mHealth app Did not use RE-AIM |
| Decreased spontaneous electrical activity in neuronal networks exposed to radiofrequency 1,800 MHz signals. | Did not address the topic Did not use mHealth app |
| Evaluation of a medication monitor-based treatment strategy for drug-sensitive tuberculosis patients in China: study protocol for a cluster randomised controlled trial. | Did not address the topic Did not use mHealth app |
| What is adverse effect of wireless local area network, using 2.45 GHz, on the reproductive system? | Did not address the topic Did not use mHealth app |
| Improving cardiometabolic health through nudging dietary behaviours and physical activity in low SES adults: design of the Supreme Nudge project. | Study Protocol |
| Effect of radiofrequency electromagnetic fields (RF-EMFS) from mobile phones on nickel release from orthodontic brackets: An in vitro study. | Did not address the topic Did not use mHealth app |
| From HIV prevention to non-communicable disease health promotion efforts in sub-Saharan Africa: A Narrative Review. | Not original article |
| Validation of the educational effectiveness of a mobile learning app to improve knowledge about MR image quality optimisation and artefact reduction. | Did not use RE-AIM |
| A randomized controlled trial of fixed- versus mobile-bearing total knee arthroplasty: a follow-up at a mean of ten years. | Did not address the topic Did not use mHealth app |
| Point of Care Solutions: Implementation of Sustainable Service Models. | Did not address the topic Did not use mHealth app |
| Effects of a depression-focused internet intervention in slot machine gamblers: A randomized controlled trial. | Did not use RE-AIM |
| Deployment of the DosiKit System Under Operational Conditions: Experience From a French Defense National Nuclear Exercise. | Did not address the topic Did not use mHealth app |
| Exposure to non-ionizing electromagnetic fields emitted from mobile phones induced DNA damage in human ear canal hair follicle cells. | Did not address the topic Did not use mHealth app |
| Failure of facet replacement system with metal-on-metal bearing surface and subsequent discovery of cobalt allergy: report of 2 cases. | Did not address the topic Did not use mHealth app |
| A smartphone app to reduce excessive alcohol consumption: Identifying the effectiveness of intervention components in a factorial randomised control trial. | Did not use RE-AIM |
| Effect of a 1800 MHz electromagnetic field emitted during embryogenesis on chick development and hatchability. | Did not address the topic Did not use mHealth app |
| Mid-term results of lateral unicondylar mobile bearing knee arthroplasty: a multicentre study of 363 cases | Did not address the topic Did not use mHealth app |
| Text4Heart II - improving medication adherence in people with heart disease: a study protocol for a randomized controlled trial. | Did not use mHealth app Did not use RE-AIM |
| Evaluation of ninety-six periprosthetic hip joint infections seen within five consecutive years. | Did not address the topic Did not use mHealth app |
| Simultaneous determination of six constituents in Yitai Capsules by HPLC | Did not address the topic Did not use mHealth app |
| Simultaneous determination of thirteen constituents in Pientzehuang by UPLC-QQQ-MS | Did not address the topic Did not use mHealth app |
| Determination of 74 new psychoactive substances in serum using automated in-line solid-phase extraction-liquid chromatography-tandem mass spectrometry. | Did not address the topic Did not use mHealth app |
| Paediatric fracture clinic re-design: Incorporating a virtual fracture clinic. | Did not address the topic Did not use mHealth app |
| Activity Trackers Implement Different Behavior Change Techniques for Activity, Sleep, and Sedentary Behaviors. | Did not address the topic Did not use mHealth app |
| Quality of life after resection of a chordoma of the mobile spine. | Did not address the topic Did not use mHealth app |
| Facing unemployment: study protocol for the implementation and evaluation of a community-based intervention for psychological well-being promotion. | Did not address the topic Did not use mHealth app |
| Reducing the rate and duration of Re-ADMISsions among patients with unipolar disorder and bipolar disorder using smartphone-based monitoring and treatment - the RADMIS trials: study protocol for two randomized controlled trials. | Did not address the topic Did not use mHealth app |
| "You Sort of Go Down a Rabbit Hole...You're Just Going to Keep on Searching": A Qualitative Study of Searching Online for Pregnancy-Related Information During Pregnancy. | Did not address the topic Did not use mHealth app |
| Long-term stability of splinted anterior mandibular teeth during supportive periodontal therapy. | Did not address the topic Did not use mHealth app |
| Acute effects of radiofrequency electromagnetic field emitted by mobile phone on brain function. | Did not address the topic Did not use mHealth app |
| Age-Related Outcome of Mobile-Bearing Total Ankle Replacement. | Did not address the topic Did not use mHealth app |
| Saúde vocal e mHealth: novas alternativas para antigos cenários | Did not use RE-AIM |
| Quantitative changes in testicular structure and function in rat exposed to mobile phone radiation. | Did not address the topic Did not use mHealth app |
| An evaluation of the telehealth facilitation of diabetes and cardiovascular care in remote Australian Indigenous communities: - protocol for the telehealth eye and associated medical services network [TEAMSnet] project, a pre-post study design. | Did not use mHealth app Did not use RE-AIM |
| Long-term exposure to 835 MHz RF-EMF induces hyperactivity, autophagy and demyelination in the cortical neurons of mice. | Did not address the topic Did not use mHealth app |
| Evaluation of 1031 primary titanium nitride coated mobile bearing total knee arthroplasties in an orthopedic clinic. | Did not address the topic Did not use mHealth app |
| Investigating factors for increased gonorrhoea re-infection in men who have sex with men attending a genitourinary clinic: a qualitative study. | Did not address the topic Did not use mHealth app |
| Review: Weak radiofrequency radiation exposure from mobile phone radiation on plants. | Not original article |
| ADAR1 restricts LINE-1 retrotransposition. | Did not address the topic Did not use mHealth app |
| Mid-term results for metaphyseal sleeves in revision knee surgery. | Did not address the topic Did not use mHealth app |
| Genomic cartography and proposal of nomenclature for the repeated, interspersed elements of the Leishmania major SIDER2 family and identification of SIDER2-containing transcripts. | Did not address the topic Did not use mHealth app |
| Simultaneous determination of eight constituents in Tiantai No.1 Tablets by HPLC-ELSD | Did not address the topic Did not use mHealth app |
| Optimizing LED lighting for space plant growth unit: Joint effects of photon flux density, red to white ratios and intermittent light pulses. | Did not address the topic Did not use mHealth app |
| A clinical audit of anatomical side marker use in a paediatric medical imaging department. | Did not address the topic Did not use mHealth app |
| Combined unicompartmental knee arthroplasty and anterior cruciate ligament reconstruction in knees with osteoarthritis and deficient anterior cruciate ligament. | Did not address the topic Did not use mHealth app |
| Survivorship and clinical outcome of the minimally invasive Uniglide medial fixed bearing, all-polyethylene tibia, unicompartmental knee arthroplasty at a mean follow-up of 7.3years. | Did not address the topic Did not use mHealth app |
| Monitoring and Follow-up of Chronic Heart Failure: a Literature Review of eHealth Applications and Systems. | Not original article |
| Retrospective 5-Year Analysis of Revision Rate and Functional Outcome of TKA With and Without Patella Implant. | Did not address the topic Did not use mHealth app |
| Open wedge high tibial osteotomy (HTO) versus mobile bearing unicondylar medial joint replacement: five years results. | Did not address the topic Did not use mHealth app |
| An e-health strategy to facilitate care of breast cancer survivors: A pilot study. | Did not use mHealth app Did not use RE-AIM |
| You're never making just one decision': exploring the lived experiences of ambulance Emergency Operations Centre personnel. | Did not address the topic Did not use mHealth app |
| Implementation of an intraoperative electron radiotherapy in vivo dosimetry program. | Did not address the topic Did not use mHealth app |
| Intraoperative 3D imaging in the treatment of elbow fractures--a retrospective analysis of indications, intraoperative revision rates, and implications in 36 cases. | Did not address the topic Did not use mHealth app |
| A Comparison Between Non-Descent Vaginal Hysterectomy and Total Abdominal Hysterectomy. | Did not address the topic Did not use mHealth app |
| Low-energy light bulbs, computers, tablets and the blue light hazard. | Did not address the topic Did not use mHealth app |
| Efficient Verification of Holograms Using Mobile Augmented Reality. | Did not address the topic Did not use mHealth app |
| Different methods for evaluating the effects of microwave radiation exposure on the nervous system. | Did not address the topic Did not use mHealth app |
| Electromagnetic field and brain development. | Did not address the topic Did not use mHealth app |
| Development and Validation of a Simple and Rapid UPLC-MS Assay for Valproic Acid and Its Comparison With Immunoassay and HPLC Methods. | Did not address the topic Did not use mHealth app |
| An HPLC method for the determination of a novel anti-hypertension agent 6,7-dimethoxy-3-(4-(4-fluorobenzyloxy)-3-methoxyphenylmethyl)quinazolin-4(3H)-one in rat plasma: application to pharmacokinetic study. | Did not address the topic Did not use mHealth app |
| Mobile phone radiation during pubertal development has no effect on testicular histology in rats. | Did not address the topic Did not use mHealth app |
| Morphological and antioxidant impairments in the spinal cord of male offspring rats following exposure to a continuous 900MHz electromagnetic field during early and mid-adolescence. | Did not address the topic Did not use mHealth app |
| he EPIC Kids Study: a randomized family-focused YMCA-based intervention to prevent type 2 diabetes in at-risk youth. | Study Protocol |
| Protocol for a systematic review of telephone delivered psychosocial interventions on relapse prevention, adherence to psychiatric medication and health risk behaviours in adults with a psychotic disorder. | Not original article |
| Memory performance, wireless communication and exposure to radiofrequency electromagnetic fields: A prospective cohort study in adolescents. | Did not address the topic Did not use mHealth app |
| [Efficacy of Early Surgical Strategy for Active Infective Endocarditis]. | Did not address the topic Did not use mHealth app |
| Re-Identification Risk versus Data Utility for Aggregated Mobility Research Using Mobile Phone Location Data. | Did not address the topic Did not use mHealth app |
| Development and feasibility testing of an education program to improve knowledge and self-care among Aboriginal and Torres Strait Islander patients with heart failure. | Did not use mHealth app Did not use RE-AIM |
| Randomised controlled trial of a mobile phone infant resuscitation guide. | Did not address the topic Did not use mHealth app |
| Concurrent validity and reliability of a novel wireless inertial measurement system to assess trunk movement. | Did not address the topic Did not use mHealth app |
| Effect of mobile phone use on metal ion release from fixed orthodontic appliances. | Did not address the topic Did not use mHealth app |
| Development of a simple and sensitive HPLC-UV method for the simultaneous determination of cannabidiol and Δ(9)-tetrahydrocannabinol in rat plasma. | Did not address the topic Did not use mHealth app |
| Biomarkers in volunteers exposed to mobile phone radiation. | Did not address the topic Did not use mHealth app |
| The effects of 2100-MHz radiofrequency radiation on nasal mucosa and mucociliary clearance in rats. | Did not address the topic Did not use mHealth app |
| Analysis on the effect of the distances and inclination angles between human head and mobile phone on SAR. | Did not address the topic Did not use mHealth app |
| Effects of chronic exposure to electromagnetic waves on the auditory system. | Did not address the topic Did not use mHealth app |
| Structural and ultrastructural study of rat liver influenced by electromagnetic radiation. | Did not address the topic Did not use mHealth app |
| Effects of 2.4 GHz radiofrequency radiation emitted from Wi-Fi equipment on microRNA expression in brain tissue. | Did not address the topic Did not use mHealth app |
| Home-based telehealth hospitalization for exacerbation of chronic obstructive pulmonary disease: findings from "the virtual hospital" trial. | Did not address the topic Did not use RE-AIM |
| The effect of exposure of rats during prenatal period to radiation spreading from mobile phones on renal development. | Did not address the topic Did not use mHealth app |
| Radiofrequency signal affects alpha band in resting electroencephalogram. | Did not address the topic Did not use mHealth app |
| The effects of N-acetylcysteine and epigallocatechin-3-gallate on liver tissue protein oxidation and antioxidant enzyme levels after the exposure to radiofrequency radiation. | Did not address the topic Did not use mHealth app |
| Seeing the forest through the trees: uncovering phenomic complexity through interactive network visualization. | Did not address the topic Did not use mHealth app |
| Ground- and satellite-based evidence of the biophysical mechanisms behind the greening Sahel. | Did not address the topic Did not use mHealth app |
| The effect of 900 and 1800 MHz GSM-like radiofrequency irradiation and nicotine sulfate administration on the embryonic development of Xenopus laevis. | Did not address the topic Did not use mHealth app |
| The effects of long-term exposure to a 2450 MHz electromagnetic field on growth and pubertal development in female Wistar rats. | Did not address the topic Did not use mHealth app |
| Effect of long-term exposure of 2.4 GHz radiofrequency radiation emitted from Wi-Fi equipment on testes functions. | Did not address the topic Did not use mHealth app |
| A prospective, randomized, controlled study using OsseoSpeed™ implants placed in maxillary fresh extraction socket: soft tissues response. | Did not address the topic Did not use mHealth app |
| Offending outcomes of a mental health youth diversion pilot scheme in England. | Did not address the topic Did not use mHealth app |
| Intracranial hemorrhage: frequency, location, and risk factors identified in a TeleStroke network. | Did not address the topic Did not use mHealth app |
| A Bridging Opportunities Work-frame to develop mobile applications for clinical decision making. | Did not use RE-AIM |
| Brain Topography of Emf-Induced Eeg-Changes in Restful Wakefulness: Tracing Current Effects, Targeting Future Prospects. | Not original article |
| Simultaneous determination of clomipramine and four benzodiazepines in human plasma by HPLC-DAD after solid phase extraction | Did not address the topic Did not use mHealth app |
| Vailidation of a LC-MS/MS method for quantification of ibuprofen enantiomers in Beagle dog plasma | Did not address the topic Did not use mHealth app |
| Cell phone radiation effects on cytogenetic abnormalities of oral mucosal cells | Did not address the topic Did not use mHealth app |
| Chronic Heart Failure Follow-up Management Based on Agent Technology | Did not address the topic Did not use mHealth app |
| Simultaneous quantitative determination of five alkaloids in Catharanthus roseus by HPLC-ESI-MS/MS. | Did not address the topic Did not use mHealth app |
| Uso de las nuevas tecnologías y telemedicina en el seguimiento del recién nacido sano | Did not address the topic Did not use mHealth app |
| Diverse radiofrequency sensitivity and radiofrequency effects of mobile or cordless phone near fields exposure in Drosophila melanogaster. | Did not address the topic Did not use mHealth app |
| Outcomes of a partnered facilitation strategy to implement primary care-mental health. | Did not use mHealth app Did not use RE-AIM |
| Design of telehealth trials--introducing adaptive approaches. | Did not address the topic Did not use mHealth app |
| Effect of electromagnetic radiations on neurodegenerative diseases- technological revolution as a curse in disguise. | Did not address the topic Did not use mHealth app |
| Intermediate frequency magnetic field generated by a wireless power transmission device does not cause genotoxicity in vitro. | Did not address the topic Did not use mHealth app |
| Evaluation of oxidant stress and antioxidant defense in discrete brain regions of rats exposed to 900 MHz radiation. | Did not address the topic Did not use mHealth app |
| Outcomes for revision total knee replacement after unicompartmental knee replacement. | Did not address the topic Did not use mHealth app |
| Impact of 60-GHz millimeter waves and corresponding heat effect on endoplasmic reticulum stress sensor gene expression. | Did not address the topic Did not use mHealth app |
| Clinical and radiological results of patients treated with orthogonal double plating for periprosthetic femoral fractures. | Did not address the topic Did not use mHealth app |
| Ten year survivorship after cemented and uncemented medial Uniglide® unicompartmental knee arthroplasties. | Did not address the topic Did not use mHealth app |
| Desenvolvimento de método indicativo de estabilidade para o antineoplásico cloridrato de doxorrubicina e avaliação da toxicidade in vitro de seus principais produtos de degradação | Did not address the topic Did not use mHealth app |
| The influence of direct mobile phone radiation on sperm quality. | Did not address the topic Did not use mHealth app |
| The use of social media among adolescents in Dar es Salaam and Mtwara, Tanzania. | Did not address the topic Did not use mHealth app |
| Behavior change techniques in top-ranked mobile apps for physical activity. | Did not use RE-AIM |
| Alteration of glycine receptor immunoreactivity in the auditory brainstem of mice following three months of exposure to radiofrequency radiation at SAR 4.0 W/kg. | Did not address the topic Did not use mHealth app |
| Midterm outcomes of total cervical total disc replacement with Bryan prosthesis. | Did not address the topic Did not use mHealth app |
| Rationale for a home dialysis virtual ward: design and implementation. | Did not address the topic Did not use mHealth app |
| The mid-term outcomes of the Oxford Domed Lateral unicompartmental knee replacement. | Did not address the topic Did not use mHealth app |
| Play and heal: randomized controlled trial of Ditto™ intervention efficacy on improving re-epithelialization in pediatric burns. | Did not use RE-AIM |
| EurOOHnet-the European research network for out-of-hours primary health care. | Did not address the topic Did not use mHealth app |
| Motion-related resource allocation in dynamic wireless visual sensor network environments. | Did not address the topic Did not use mHealth app |
| A UPLC-MS/MS method for in vivo and in vitro pharmacokinetic studies of psoralenoside, isopsoralenoside, psoralen and isopsoralen from Psoralea corylifolia extract. | Did not address the topic Did not use mHealth app |
| Assessment of oxidant/antioxidant status in saliva of cell phone users. | Did not address the topic Did not use mHealth app |
| Introduction of the I-gel supraglottic airway device for prehospital airway management in a UK ambulance service. | Did not address the topic Did not use mHealth app |
| Simultaneous quantitative determination of five alkaloids in Catharanthus roseus by HPLC-ESI-MS/MS | Did not address the topic Did not use mHealth app |
| Fast and delayed locomotor response to acute high-dose nicotine administration in adult male rats. | Did not address the topic Did not use mHealth app |
| Long-term follow-up of mobile-bearing total ankle replacement in patients with inflammatory joint disease. | Did not address the topic Did not use mHealth app |
| Evaluation of the effects of mobile phones on the neural tube development of chick embryos. | Did not address the topic Did not use mHealth app |
| Overproduction of free radical species in embryonal cells exposed to low intensity radiofrequency radiation. | Did not address the topic Did not use mHealth app |
| Recent reports of Wi-Fi and mobile phone-induced radiation on oxidative stress and reproductive signaling pathways in females and males. | Did not address the topic Did not use mHealth app |
| Management of scientific information with Google Drive. | Did not address the topic Did not use mHealth app |
| Effect of 900MHz electromagnetic fields emitted from cellular phones on fracture healing: an experimental study on rats. | Did not address the topic Did not use mHealth app |
| Case-control study of the association between malignant brain tumours diagnosed between 2007 and 2009 and mobile and cordless phone use. | Did not address the topic Did not use mHealth app |
| The effect of pulsed electromagnetic radiation from mobile phone on the levels of monoamine neurotransmitters in four different areas of rat brain. | Did not address the topic Did not use mHealth app |
| An isolated penile mass in a young adult turned out to be a primary marginal zone lymphoma of the penis. A case report and a review of literature. | Not original article |
| Effects of exposure to electromagnetic field radiation (EMFR) generated by activated mobile phones on fasting blood glucose. | Did not address the topic Did not use mHealth app |
| Modulation of wireless (2.45 GHz)-induced oxidative toxicity in laryngotracheal mucosa of rat by melatonin. | Did not address the topic Did not use mHealth app |
| Stimulation of the brain with radiofrequency electromagnetic field pulses affects sleep-dependent performance improvement. | Did not address the topic Did not use mHealth app |
| Clinical indication for intraoperative 3D imaging during open reduction of fractures of the mandibular angle. | Did not address the topic Did not use mHealth app |
| Early results of a domed tibia, mobile bearing lateral unicompartmental knee arthroplasty from an independent centre. | Did not address the topic Did not use mHealth app |
| Health care in small prisons: incorporating high-quality standards. | Did not address the topic Did not use mHealth app |
| The toxic effects of mobile phone radiofrequency (940 MHz) on the structure of calf thymus DNA. | Did not address the topic Did not use mHealth app |
| Prospective effects of traumatic event re-exposure and post-traumatic stress disorder in syringe exchange participants. | Did not address the topic Did not use mHealth app |
| Protocol for "Seal or Varnish?" (SoV) trial: a randomised controlled trial to measure the relative cost and effectiveness of pit and fissure sealants and fluoride varnish in preventing dental decay. | Did not address the topic Did not use mHealth app |
| Seasonal variation of the ¹³7Cs level and its relationship with potassium and carbon levels in conifer needles. | Did not address the topic Did not use mHealth app |
| Recruiting and engaging new mothers in nutrition research studies: lessons from the Australian NOURISH randomised controlled trial. | Did not address the topic Did not use mHealth app |
| Effects of GSM 900 MHz on middle cerebral artery blood flow assessed by transcranial Doppler sonography. | Did not address the topic Did not use mHealth app |
| LINE-1 retrotransposition events regulate gene expression after X-ray irradiation. | Did not address the topic Did not use mHealth app |
| Effects of simultaneous combined exposure to CDMA and WCDMA electromagnetic field on immune functions in rats. | Did not address the topic Did not use mHealth app |
| Rat testicular impairment induced by electromagnetic radiation from a conventional cellular telephone and the protective effects of the antioxidants vitamins C and E | Did not address the topic Did not use mHealth app |
| The effects of long-term exposure of magnetic field via 900-MHz GSM radiation on some biochemical parameters and brain histology in rats. | Did not address the topic Did not use mHealth app |
| The effect of melatonin on body mass and behaviour of rats during an exposure to microwave radiation from mobile phone. | Did not address the topic Did not use mHealth app |
| Exposure to radiofrequency electromagnetic fields and sleep quality: a prospective cohort study. | Did not address the topic Did not use mHealth app |
| Total ankle arthroplasty - three-component total ankle arthroplasty in western France: a radiographic study. | Did not address the topic Did not use mHealth app |
| Changes in tympanic temperature during the exposure to electromagnetic fields emitted by mobile phone. | Did not address the topic Did not use mHealth app |
| Human short-term exposure to electromagnetic fields emitted by mobile phones decreases computer-assisted visual reaction time. | Did not address the topic Did not use mHealth app |
| 900 MHz radiation does not induce micronucleus formation in different cell types. | Did not address the topic Did not use mHealth app |
| Change in working alliance and recovery in severe mental illness: an exploratory study. | Did not address the topic Did not use mHealth app |
| The influence of microwave radiation from cellular phone on fetal rat brain. | Did not address the topic Did not use mHealth app |
| Evaluation of radiation during EVAR performed on a mobile C-arm. | Did not address the topic Did not use mHealth app |
| The use of FDTD in establishing in vitro experimentation conditions representative of lifelike cell phone radiation on the spermatozoa. | Did not address the topic Did not use mHealth app |
| Mobile health IT: the effect of user interface and form factor on doctor-patient communication. | Did not use mHealth app Did not use RE-AIM |
| Assessment of intermittent UMTS electromagnetic field effects on blood circulation in the human auditory region using a near-infrared system. | Did not address the topic Did not use mHealth app |
| The genotoxic effect of radiofrequency waves on mouse brain. | Did not address the topic Did not use mHealth app |
| Effects of the exposure to mobile phones on male reproduction: a review of the literature. | Not original article |
| LCS mobile bearing total knee arthroplasty without patellar resurfacing. Does the unresurfaced patella affect outcome? Survivorship at a minimum 10-year follow-up. | Did not address the topic Did not use mHealth app |
| Thermal effects of mobile phone RF fields on children: a provocation study. | Did not address the topic Did not use mHealth app |
| The effect of short messaging service text on non-attendance in a general ophthalmology clinic. | Did not use mHealth app Did not use RE-AIM |
| Quiet eye training facilitates competitive putting performance in elite golfers. | Did not address the topic Did not use mHealth app |
| False-positive mammographic screening: factors influencing re-attendance over a decade of screening | Did not address the topic Did not use mHealth app |
| Orientation and detachment dynamics of Bacillus spores from stainless steel under controlled shear flow: modelling of the adhesion force. | Did not address the topic Did not use mHealth app |
| Prenatal cell phone use and developmental milestone delays among infants. | Did not address the topic Did not use mHealth app |
| Novel use of an air-filled breast prosthesis to allow radiotherapy to recurrent colonic cancer. | Did not address the topic Did not use mHealth app |
| Cognitive performance measures in bioelectromagnetic research--critical evaluation and recommendations. | Did not address the topic Did not use mHealth app |
| The connection between agr and SCCmec elements of Staphylococcus aureus strains and their response to photodynamic inactivation. | Did not address the topic Did not use mHealth app |
| Open reduction and internal fixation of a traumatic diastasis of the pubic symphysis: one-year radiological and functional outcomes. | Did not address the topic Did not use mHealth app |
| A second decade lifetable survival analysis of the Oxford unicompartmental knee arthroplasty. | Did not address the topic Did not use mHealth app |
| Electric and magnetic fields do not modify the biochemical properties of FRTL-5 cells. | Did not address the topic Did not use mHealth app |
| Jigsaw: engaging communities in the development and implementation of youth mental health services and supports in the Republic of Ireland. | Did not address the topic Did not use mHealth app |
| Do mobile phone base stations affect sleep of residents? Results from an experimental double-blind sham-controlled field study. | Did not address the topic Did not use mHealth app |
| Effects of GSM signals during exposure to event related potentials (ERPs). | Did not address the topic Did not use mHealth app |
| [HPLC-ELSD fingerprint and chemical constituents of Rhizoma Panacis Japonici]. | Did not address the topic Did not use mHealth app |
| Comparative study on skin dose measurement using MOSFET and TLD for pediatric patients with acute lymphatic leukemia. | Did not address the topic Did not use mHealth app |
| Combined effects of 872 MHz radiofrequency radiation and ferrous chloride on reactive oxygen species production and DNA damage in human SH-SY5Y neuroblastoma cells. | Did not address the topic Did not use mHealth app |
| Modified repair in patients with Ebstein's anomaly. | Did not address the topic Did not use mHealth app |
| Can a health forecasting service offer COPD patients a novel way to manage their condition? | Did not address the topic Did not use mHealth app |
| Principal component analysis of the P600 waveform: RF and gender effects. | Did not address the topic Did not use mHealth app |
| Immigrant women's experiences of receiving care in a mobile health clinic. | Did not address the topic Did not use mHealth app |
| Non-invasive bleaching of the human lens by femtosecond laser photolysis. | Did not address the topic Did not use mHealth app |
| A new rating instrument to assess festination and freezing gait in Parkinsonian patients. | Did not address the topic Did not use mHealth app |
| Absence of short-term effects of UMTS exposure on the human auditory system. | Did not address the topic Did not use mHealth app |
| Microglial activation as a measure of stress in mouse brains exposed acutely (60 minutes) and long-term (2 years) to mobile telephone radiofrequency fields. | Did not address the topic Did not use mHealth app |
| The influence of 1800 MHz GSM-like signals on hepatic oxidative DNA and lipid damage in nonpregnant, pregnant, and newly born rabbits. | Did not address the topic Did not use mHealth app |
| Clinical and radiological outcomes of fixed- versus mobile-bearing total knee replacement: a meta-analysis. | Not original article |
| HPLC-ELSD fingerprint and chemical constituents of Rhizoma Panacis Japonici | Did not address the topic Did not use mHealth app |
| Determination of ginsenoside Rb_1,Re,Rb_2 and Rd in Shenqi Granules by microbore liquid chromatography | Did not address the topic Did not use mHealth app |
| Effect of mobile phone exposure on apoptotic glial cells and status of oxidative stress in rat brain. | Did not address the topic Did not use mHealth app |
| Cytogenetic effects of exposure to 2.3 GHz radiofrequency radiation on human lymphocytes in vitro. | Did not address the topic Did not use mHealth app |
| [A motivating experience for emergency medical services: the first Turkish Ambulance Rally]. | Did not address the topic Did not use mHealth app |
| The redesign and re-evaluation of an internet-based telerehabilitation system for the assessment of dysarthria in adults. | Did not address the topic Did not use mHealth app |
| The effect of electromagnetic radiation in the mobile phone range on the behaviour of the rat. | Did not address the topic Did not use mHealth app |
| [Distal intraarticular humerus fracture in the elderly: prosthesis or osteosynthesis?]. | Did not address the topic Did not use mHealth app |
| Translating clinical informatics interventions into routine clinical care: how can the RE-AIM framework help? | Did not use mHealth app Did not use RE-AIM |
| Mobile phones exposure induces changes of contingent negative variation in humans. | Did not address the topic Did not use mHealth app |
| An improved LC-MS/MS method for quantitative determination of ilaprazole and its metabolites in human plasma and its application to a pharmacokinetic study. | Did not address the topic Did not use mHealth app |
| Community outcomes of mentally disordered homicide offenders in Victoria. | Did not address the topic Did not use mHealth app |
| Clinical and radiological outcomes after revision to the low-contact-stress mobile-bearing total knee arthroplasty. | Did not address the topic Did not use mHealth app |
| Human fibroblasts and 900 MHz radiofrequency radiation: evaluation of DNA damage after exposure and co-exposure to 3-chloro-4-(dichloromethyl)-5-hydroxy-2(5h)-furanone (MX). | Did not address the topic Did not use mHealth app |
| Radioprotective effects of honeybee venom (Apis mellifera) against 915-MHz microwave radiation-induced DNA damage in wistar rat lymphocytes: in vitro study. | Did not address the topic Did not use mHealth app |
| Intermediate clinical and radiological results of cervical TDR (Mobi-C) with up to 2 years of follow-up. | Did not address the topic Did not use mHealth app |
| Exposure to an 890-MHz mobile phone-like signal and serum levels of S100B and transthyretin in volunteers. | Did not address the topic Did not use mHealth app |
| Short-term exposure to mobile phone base station signals does not affect cognitive functioning or physiological measures in individuals who report sensitivity to electromagnetic fields and controls. | Did not address the topic Did not use mHealth app |
| expression of the water channel protein, aquaporin-4, in mouse brains exposed to mobile telephone radiofrequency fields. | Did not address the topic Did not use mHealth app |
| Absence of genotoxic potential of 902 MHz (GSM) and 1747 MHz (DCS) wireless communication signals: In vivo two-year bioassay in B6C3F1 mice. | Did not address the topic Did not use mHealth app |
| Heat shock protein induction in fetal mouse brain as a measure of stress after whole of gestation exposure to mobile telephony radiofrequency fields. | Did not address the topic Did not use mHealth app |
| Disturbance of cell proliferation in response to mobile phone frequency radiation. | Did not address the topic Did not use mHealth app |
| The effects of microwave emitted by cellular phones on ovarian follicles in rats. | Did not address the topic Did not use mHealth app |
| Effects of intrauterine and extrauterine exposure to GSM-like radiofrequency on distortion product otoacoustic emissions in infant male rabbits. | Did not address the topic Did not use mHealth app |
| Extremely low-frequency magnetic fields effects on the snail single neurons. | Did not address the topic Did not use mHealth app |
| Combinative exposure effect of radio frequency signals from CDMA mobile phones and aphidicolin on DNA integrity. | Did not address the topic Did not use mHealth app |
| Usefulness of intraoperative ultra low-field magnetic resonance imaging in glioma surgery. | Did not address the topic Did not use mHealth app |
| Melatonin reduces oxidative stress induced by chronic exposure of microwave radiation from mobile phones in rat brain. | Did not address the topic Did not use mHealth app |
| The involvement of different mobile LINE copies of blood plasma and extrachromosomal DNA of liver cells in systemic adaptive response. | Did not address the topic Did not use mHealth app |
| Radiofrequency radiation does not significantly affect ornithine decarboxylase activity, proliferation, or caspase-3 activity of fibroblasts in different physiological conditions. | Did not address the topic Did not use mHealth app |
| Lifestyle and testicular dysfunction: a brief update. | Did not address the topic Did not use mHealth app |
| The influence of the call with a mobile phone on heart rate variability parameters in healthy volunteers. | Did not address the topic Did not use mHealth app |
| Epidemiology and outcome after hip fracture in the under 65s-evidence from the Scottish Hip Fracture Audit. | Did not address the topic Did not use mHealth app |
| Penile epidermal inclusion cyst: a late complication of penile girth enhancement surgery. | Did not address the topic Did not use mHealth app |
| Exposure to low level GSM 935 MHZ radiofrequency fields does not induce apoptosis in proliferating or differentiated murine neuroblastoma cells. | Did not address the topic Did not use mHealth app |
| Screening for the metabolic syndrome in community psychiatric patients prescribed antipsychotics: a quality improvement programme. | Did not address the topic Did not use mHealth app |
| Mobile phones: influence on auditory and vestibular systems. | Not original article |
| Setup and dosimetry for exposing anaesthetised pigs in vivo to 900 MHz GSM mobile phone fields. | Did not address the topic Did not use mHealth app |
| Telefones celulares: influência nos sistemas auditivo e vestibular: [revisão] | Not original article |
| Setup and dosimetry for exposure of human skin in vivo to RF-EMF at 900 MHz. | Did not address the topic Did not use mHealth app |
| What happens to patients seen only once by psychiatric services? Findings from a follow-up study. | Did not address the topic Did not use mHealth app |
| Effects of GSM 1800 MHz on dendritic development of cultured hippocampal neurons. | Did not address the topic Did not use mHealth app |
| Short GSM mobile phone exposure does not alter human auditory brainstem response. | Did not address the topic Did not use mHealth app |
| Micronucleus frequency in erythrocytes of mice after long-term exposure to radiofrequency radiation. | Did not address the topic Did not use mHealth app |
| Re-hospitalization of first-in-life admitted schizophrenic patients before and after rehabilitation legislation: a comparison of two national cohorts. | Did not address the topic Did not use mHealth app |
| Studying gene expression profile of rat neuron exposed to 1800MHz radiofrequency electromagnetic fields with cDNA microassay. | Did not address the topic Did not use mHealth app |
| Mental health. Order in the house. | Did not use mHealth app Did not use RE-AIM |
| Formation of reactive oxygen species in L929 cells after exposure to 900 MHz RF radiation with and without co-exposure to 3-chloro-4-(dichloromethyl)-5-hydroxy-2(5H)-furanone. | Did not address the topic Did not use mHealth app |
| Neurobehavioral effects among inhabitants around mobile phone base stations. | Did not address the topic Did not use mHealth app |
| No apoptosis is induced in rat cortical neurons exposed to GSM phone fields. | Did not address the topic Did not use mHealth app |
| Influence of electromagnetic fields and protective effect of CAPE on bone mineral density in rats. | Did not address the topic Did not use mHealth app |
| Exposure of cultured astroglial and microglial brain cells to 900 MHz microwave radiation. | Did not address the topic Did not use mHealth app |
| Adopting a personal digital assistant system: application of Lewin's change theory. | Did not address the topic Did not use mHealth app |
| The role of telementoring and telerobotic assistance in the provision of laparoscopic colorectal surgery in rural areas. | Did not address the topic Did not use mHealth app |
| [Simultaneous determination of ephedrine and chlorpheniramine in human plasma by a highly sensitive liquid chromatography-tandem mass spectrometric method]. | Did not address the topic Did not use mHealth app |
| Microwave exposure of neuronal cells in vitro: Study of apoptosis. | Did not address the topic Did not use mHealth app |
| Simulation of the geomagnetic field experienced by the International Space Station in its revolution around the Earth: effects on psychophysiological responses to affective picture viewing. | Did not address the topic Did not use mHealth app |
| Proliferation and apoptosis in a neuroblastoma cell line exposed to 900 MHz modulated radiofrequency field. | Did not address the topic Did not use mHealth app |
| In vitro effects of GSM modulated radiofrequency fields on human immune cells. | Did not address the topic Did not use mHealth app |
| Psychophysiological tests and provocation of subjects with mobile phone related symptoms. | Did not address the topic Did not use mHealth app |
| Remote sensing for predicting potential habitats of Oncomelania hupensis in Hongze, Baima and Gaoyou lakes in Jiangsu province, China. | Did not address the topic Did not use mHealth app |
| Simultaneous determination of ephedrine and chlorpheniramine in human plasma by a highly sensitive liquid chromatography-tandem mass spectrometric method | Did not address the topic Did not use mHealth app |
| Mobile phone-induced myocardial oxidative stress: protection by a novel antioxidant agent caffeic acid phenethyl ester. | Did not address the topic Did not use mHealth app |
| A Swedish version of the GOHAI index. Psychometric properties and validation. | Did not address the topic Did not use mHealth app |
| Electromagnetic fields from mobile phones do not affect the inner auditory system of Sprague-Dawley rats. | Did not address the topic Did not use mHealth app |
| The effect of electromagnetic waves on the growth of Entamoeba histolytica and Entamoeba dispar. | Did not address the topic Did not use mHealth app |
| Effects of 900 MHz electromagnetic fields exposure on cochlear cells' functionality in rats: evaluation of distortion product otoacoustic emissions. | Did not address the topic Did not use mHealth app |
| The impact of organ motion on intestine doses and complication probabilities in radiotherapy of bladder cancer. | Did not address the topic Did not use mHealth app |
| Oxidative damage in the kidney induced by 900-MHz-emitted mobile phone: protection by melatonin. | Did not address the topic Did not use mHealth app |
| Interaction of mobile phones with superficial passive metallic implants. | Did not address the topic Did not use mHealth app |
| Population mobility: characteristics of people registering with general practices | Did not address the topic Did not use mHealth app |
| LC-ESI-MS/MS determination of 4-hydroxy-trans-2-nonenal Michael adducts with cysteine and histidine-containing peptides as early markers of oxidative stress in excitable tissues. | Did not address the topic Did not use mHealth app |
| Toward optimizing lighting as a countermeasure to sleep and circadian disruption in space flight. | Did not address the topic Did not use mHealth app |
| [Telemedical-supported screening of retinal vessels ("talking eyes")]. | Did not address the topic Did not use mHealth app |
| Impersonal trust and professional authority: exploring the dynamics. | Did not address the topic Did not use mHealth app |
| [Effect of electromagnetic field produced by mobile phones on the activity of superoxide dismutase (SOD-1)--in vitro researches]. | Did not address the topic Did not use mHealth app |
| Effect of 900 MHz electromagnetic fields on nonthermal induction of heat-shock proteins in human leukocytes. | Did not address the topic Did not use mHealth app |
| Combined Abciximab REteplase Stent Study in acute myocardial infarction (CARESS in AMI). | Did not address the topic Did not use mHealth app |
| In vitro exposure of human lymphocytes to 900 MHz CW and GSM modulated radiofrequency: studies of proliferation, apoptosis and mitochondrial membrane potential. | Did not address the topic Did not use mHealth app |
| Fixed or mobile bearing unicompartmental knee replacement? A comparative cohort study. | Did not address the topic Did not use mHealth app |
| The effects of a transitional discharge model for psychiatric patients. | Did not address the topic Did not use mHealth app |
| Apoptosis induced by ultraviolet radiation is enhanced by amplitude modulated radiofrequency radiation in mutant yeast cells. | Did not address the topic Did not use mHealth app |
| Effect of external stresses on protein conformation: a computer modelling study. | Did not address the topic Did not use mHealth app |
| [Determination of hydromorphone in Beagle dogs plasma by liquid chromatography-tandem mass spectrometry]. | Did not address the topic Did not use mHealth app |
| Prevention of mobile phone induced skin tissue changes by melatonin in rat: an experimental study. | Did not address the topic Did not use mHealth app |
| Determination of hydromorphone in Beagle dogs plasma by liquid chromatography-tandem mass spectrometry | Did not address the topic Did not use mHealth app |
| Comparisons of peak SAR levels in concentric sphere head models of children and adults for irradiation by a dipole at 900 MHz. | Did not address the topic Did not use mHealth app |
| Effects of in vivo exposure to GSM-modulated 900 MHz radiation on mouse peripheral lymphocytes. | Did not address the topic Did not use mHealth app |
| Effects of GSM-900 microwaves on DMBA-induced mammary gland tumors in female Sprague-Dawley rats. | Did not address the topic Did not use mHealth app |
| Enhancement of plasmid curing by 9-aminoacridine and two phenothiazines in the presence of proton pump inhibitor 1-(2-benzoxazolyl)-3,3,3-trifluoro-2-propanone. | Did not address the topic Did not use mHealth app |
| Non-ionizing electromagnetic radiations, emitted by a cellular phone, modify cutaneous blood flow. | Did not address the topic Did not use mHealth app |
| Neonatal doses from X ray examinations by birth weight in a neonatal intensive care unit. | Did not address the topic Did not use mHealth app |
| Human brain wave activity during exposure to radiofrequency field emissions from mobile phones. | Did not address the topic Did not use mHealth app |
| The acute effects of exposure to the electromagnetic field emitted by mobile phones on human attention. | Did not address the topic Did not use mHealth app |
| Determination of wogonin in rat plasma by liquid chromatography-tandem mass spectrometry | Did not address the topic Did not use mHealth app |
| Prioritising referrals to a community mental health team. | Did not address the topic Did not use mHealth app |
| Displaced intracapsular neck of femur fractures in mobile independent patients: total hip replacement or hemiarthroplasty? | Did not address the topic Did not use mHealth app |
| Ethics in community psychiatry. | Did not address the topic Did not use mHealth app |
| [Contribution of telemedicine applied to digestive cancer]. | Did not use mHealth app Did not use RE-AIM |
| Complement activation by bacterial surface glycolipids: a study with planar bilayer membranes. | Did not address the topic Did not use mHealth app |
| Healing at implants with and without primary bone contact. An experimental study in dogs. | Did not address the topic Did not use mHealth app |
| No short-term effects of digital mobile radio telephone on the awake human electroencephalogram. | Did not address the topic Did not use mHealth app |
| The role of passive transbilayer drug movement in multidrug resistance and its modulation. | Did not address the topic Did not use mHealth app |
| Examiner agreement on periodontal indices during dental surveys of elders. | Did not address the topic Did not use mHealth app |
| Pathways of protein sorting and membrane traffic between the rough endoplasmic reticulum and the Golgi complex. | Did not address the topic Did not use mHealth app |
| Pre-hospital care in Barbados - abstract | Did not address the topic Did not use mHealth app |
| Experiment "Seeds" on Biokosmos 9. Dosimetric part. | Did not address the topic Did not use mHealth app |
| Determination of narigin,hesperidin and neohesperidin in Weili Tablet by HPLC | Did not address the topic Did not use mHealth app |
| Simultaneous determination of notoginsenoside R_1,ginsenoside Rg_1,ginsenoside Re,ginsenoside Rb_1 in Yunnan Baiyao Powder by RP-HPLC | Did not address the topic Did not use mHealth app |
| Determination of notoginsenoside R_1,ginsenoside Rg_1,ginsenoside Re and ginsenoside Rb_1 in Compound Danshen Tablets of different pharmaceutical factories by HPLC | Did not address the topic Did not use mHealth app |
| Determination of ginsenoside Rg1、Re and Rb1 in Tangniaole Granule by HPLC | Did not address the topic Did not use mHealth app |
| Determination of the four main saponins in total saponin of Radix et Rhizoma Notoginseng Buccal Tablets by HPLC | Did not address the topic Did not use mHealth app |
| Determination of ginsenoside Rg1 and ginsenoside Re in Wushenqi Oral Liquid by RP-HPLC | Did not address the topic Did not use mHealth app |
| Determination of ginsenoside Re、ginsenoside Rg_1 and schisandrin in Huoliyuan Tablets by HPLC | Did not address the topic Did not use mHealth app |
| Determination of four components in Shengmai Injection by HPLC | Did not address the topic Did not use mHealth app |
| Determination of ginsenoside in Yixin Capsule by HPLC | Did not address the topic Did not use mHealth app |
| Determination of ginsenoside R_e and R_(g1) in Compound Jiangtang Oral Liquid by HPLC | Did not address the topic Did not use mHealth app |
| Thermal effects and histologic changes from Nd:YAG laser irradiation on normal and diseased aortic tissue using a novel angioplasty catheter with a mobile optical fiber: an in vitro assessment. | Did not address the topic Did not use mHealth app |
| [Intensification of the fight against leprosy using early and systematic polychemotherapy]. | Did not address the topic Did not use mHealth app |
| [Infected prosthesis of large joints]. | Did not address the topic Did not use mHealth app |
| [The problem of dorsal spondylodesis while using traction rods (author's transl)]. | Did not address the topic Did not use mHealth app |
| A prevalence study of oral mucosal lesions in an adult Swedish population. | Did not address the topic Did not use mHealth app |
| Cell to cell interaction in the immune response. V. Target cells for tolerance induction. | Did not address the topic Did not use mHealth app |
| Implementation evaluation of a teledermatology virtual clinic at an academic medical center. | Did not use mHealth app Did not use RE-AIM |
| A Rapid-Learning Health System to Support Implementation of Early Intervention Services for Psychosis in Quebec, Canada: Protocol. | Study Protocol |
| Protocol for a process evaluation: face-to-face physiotherapy compared with a supported home exercise programme for the management of musculoskeletal conditions: the REFORM trial. | Study Protocol |
| Protocol for process evaluation of SMART Mental Health cluster randomised control trial: an intervention for management of common mental disorders in India. | Study Protocol |
| A digital mental health intervention to reduce depressive symptoms among overseas Filipino workers: protocol for a pilot hybrid type 1 effectiveness-implementation randomized controlled trial. | Study Protocol |
| Implementing Video to Home to Increase Access to Evidence-Based Psychotherapy for Rural Veterans. | Did not use mHealth app Did not use RE-AIM |
| Dissemination and Implementation of a Google Apple Exposure Notification System for COVID-19 Risk Mitigation at a National Public University: Protocol for a Pilot Evaluation Study in a Real-World Setting. | Did not address the topic Did not use mHealth app |
| An integrated community health worker intervention in rural Nepal: a type 2 hybrid effectiveness-implementation study protocol. | Did not use mHealth app Did not use RE-AIM |
| Evaluation of a multidisciplinary lipid clinic to improve the care of individuals with severe lipid conditions: a RE-AIM framework analysis. | Did not use mHealth app Did not use RE-AIM |
| Evaluation of a Healthy Relationship Smartphone App With Indigenous Young People: Protocol for a Co-designed Stepped Wedge Randomized Trial. | Study Protocol |
| Barriers and facilitators in implementing a pilot, pragmatic, telemedicine-delivered healthy lifestyle program for obesity management in a rural, academic obesity clinic. | Did not use mHealth app Did not use RE-AIM |
| Implementation of an evidence-based tobacco control intervention for school teachers in India: Evaluating the effects of a capacity-building strategy. | Did not use mHealth app Did not use RE-AIM |
| HOMBRE: A randomized controlled trial to compare two approaches to weight loss for overweight and obese Latino men (Hombres con Opciones para Mejorar el Bienestar y bajar el Riesgo de Enfermedades crónicas; men with choices to improve well-being and decrease chronic disease risk). | Study Protocol |
| App-Delivered Self-Management Intervention Trial selfBACK for People With Low Back Pain: Protocol for Implementation and Process Evaluation. | Study Protocol |
| Virtual Family-Centered Rounds in the Neonatal Intensive Care Unit: Protocol for a Cluster Randomized Controlled Trial. | Study Protocol |
| Evaluating the efficacy of the HITSystem 2.1 to improve PMTCT retention and maternal viral suppression in Kenya: Study protocol of a cluster-randomized trial. | Study Protocol |
| Implementation of Video Visits During COVID-19: Lessons Learned From a Primary Care Practice in New York City. | Did not use mHealth app Did not use RE-AIM |
| The therapist's role in the implementation of internet-based cognitive behavioural therapy for patients with depression: study protocol. | Study Protocol |
| Application of the ConNECT Framework to achieve digital health equity | Did not use mHealth app Did not use RE-AIM |
| Reach Outcomes and Costs of Different Physician Referral Strategies for a Weight Management Program Among Rural Primary Care Patients: Type 3 Hybrid Effectiveness-Implementation Trial | Did not use mHealth app Did not use RE-AIM |
| Clinical decision support systems for chronic obstructive pulmonary disease (COPD) in hospitals: A systematic review | Not original article |
| Older Adult Mental Health: A Student-Led Project to Reduce Stigma Among Residents Living in a Publicly Supported Housing | Did not address the topic Did not use mHealth app |
| Implementation of eLearning solutions for patients with chronic pain conditions | Did not use RE-AIM |
| Preventing Frail and Elderly Hospital Admissions: Developing an Evaluation Framework for the 'Closer to Home' Quality Improvement Programme in NHS Forth Valley | Did not use mHealth app Did not use RE-AIM |
| Get waivered remote: Nationwide, remote DEA-x waiver course in response to COVID-19 | Did not address the topic Did not use mHealth app |
| Patient and provider perspectives on the design and implementation of an electronic consultation system for kidney care delivery in Canada: a focus group study | Did not use mHealth app Did not use RE-AIM |
| Dissemination of a telehealth cardiovascular risk service: The CVRS live protocol | Did not use mHealth app Did not use RE-AIM |
| Dissemination of a telehealth cardiovascular risk service: The CVRS live protocol | Did not address the topic Did not use mHealth app |
| Integration of Mobile Health Into Sickle Cell Disease Care to Increase Hydroxyurea Utilization: Protocol for an Efficacy and Implementation Study | Study Protocol |
| A Suicide Prevention Intervention for Emerging Adult Sexual and Gender Minority Groups: Protocol for a Pilot Hybrid Effectiveness Randomized Controlled Trial | Did not address the topic Did not use mHealth app |
| Effectiveness of an mHealth intervention to increase adherence to triage of HPV DNA positive women who have performed self-collection (the ATICA study): A hybrid type I cluster randomised effectiveness-implementation trial | Did not use mHealth app Did not use RE-AIM |
| "Blood pressure monitoring should be a habit": adaptation of the Check. Change. Control. program for Asian American older adults, from group-based in-person to one-on-one telephone delivery | Did not use mHealth app Did not use RE-AIM |
| Systematic review of alternative HIV preexposure prophylaxis care delivery models to improve preexposure prophylaxis services | Not original article |
| Perceived Benefits, Barriers, and Facilitators of a Digital Patient-Reported Outcomes Tool for Routine Diabetes Care: Protocol for a National, Multicenter, Mixed Methods Implementation Study | Study Protocol |
| Application and effectiveness of eHealth strategies for metabolic and bariatric surgery patients: A systematic review | Not original article |
| Text Messaging Versus Email Messaging to Support Patients With Major Depressive Disorder: Protocol for a Randomized Hybrid Type II Effectiveness-Implementation Trial | Did not use mHealth app Did not use RE-AIM |
| Development of a Health Information Technology Tool for Behavior Change to Address Obesity and Prevent Chronic Disease Among Adolescents: Designing for Dissemination and Sustainment Using the ORBIT Model | Did not use RE-AIM |
| Using a Tailored Digital Health Intervention for Family Communication and Cascade Genetic Testing in Swiss and Korean Families With Hereditary Breast and Ovarian Cancer: Protocol for the DIALOGUE Study | Did not use RE-AIM |
| Leveraging Community Health Workers and a Responsive Digital Health System to Improve Vaccination Coverage and Timeliness in Resource-Limited Settings: Protocol for a Cluster Randomized Type 1 Effectiveness-Implementation Hybrid Study | Study Protocol |
| Leveraging Community Health Workers and a Responsive Digital Health System to Improve Vaccination Coverage and Timeliness in Resource-Limited Settings: Protocol for a Cluster Randomized Type 1 Effectiveness-Implementation Hybrid Study | Case Report |
| Patient Acceptability of Symptom Screening and Patient Education Using a Chatbot for Autoimmune Inflammatory Diseases: Survey Study | Did not use mHealth app Did not use RE-AIM |
| Study protocol for the Screen-Free Time with Friends Feasibility Trial | Did not use mHealth app Did not use RE-AIM |
| Assessing equity in the uptake of remote foot temperature monitoring in a large integrated US healthcare system | Did not use mHealth app Did not use RE-AIM |
| Implementation of COPD Clinical Practice Guidelines with Use of Telehealth | Did not use mHealth app Did not use RE-AIM |
| Implementation evaluation of tiered tele-triage pathways for burn center consultations and transfers | Did not use mHealth app Did not use RE-AIM |
| Strategies and Best Practices That Enhance the Physical Activity Levels of Undergraduate University Students: A Systematic Review | Not original article |
| Accelerating implementation of adolescent digital health prevention programs: analysis of insights from Australian stakeholders | Did not use mHealth app Did not use RE-AIM |
| A community-based intervention to improve screening, referral and follow-up of non-communicable diseases and anaemia amongst pregnant and postpartum women in rural India: study protocol for a cluster randomised trial | Study Protocol |
| Descriptive Report of a Pharmacist-Directed Preconception Care Outreach Program in a Rural Maternity Care Desert | Did not use mHealth app Did not use RE-AIM |
| The NASSS (Non-Adoption, Abandonment, Scale-Up, Spread and Sustainability) framework use over time: A scoping review | Not original article |
| Older Adult Mental Health: A Student-Led Project to Reduce Stigma Among Residents Living in a Publicly Supported Housing | Not original article |
| Telehealth Use in a National Pediatric Weight Management Sample During the COVID-19 Pandemic | Did not use mHealth app Did not use RE-AIM |
| IS INFLUENZ-ER PROGRAM FEASIBLE AND SAFE? ASSESSMENT OF HOSPITAL STAFF ACCEPTABILITY AND UTILISATION OF A TELEMEDICINE-SUPPORTED EARLY DISCHARGE PROGRAM | Did not use mHealth app Did not use RE-AIM |
| Telerehabilitation in the 'real-world': Implementation of remotely delivered pulmonary rehabilitation | Did not address the topic Did not use mHealth app |
| Outcomes of a risk assessment and management program using telecare consultation among patients with diabetes mellitus in general out-patient clinic: a hybrid effectiveness-implementation study protocol | Study Protocol |
| Structured evaluation of a virtual emergency department triage model of care: A study protocol | Did not use mHealth app Did not use RE-AIM |
| Implementation of a threefold intervention to improve palliative care for persons experiencing homelessness: a process evaluation using the RE-AIM framework | Did not use mHealth app Did not use RE-AIM |
| Optimising implementation of telehealth in oncology: A systematic review examining barriers and enablers using the RE-AIM planning and evaluation framework | Not original article |
| Implementation of a colorectal cancer screening intervention in Malaysia (CRC-SIM) in the context of a pandemic: Study protocol | Did not use mHealth app Did not use RE-AIM |
| Implementation of a telehealth videoconference to improve hospital-to-skilled nursing care transitions: Preliminary data | Did not use mHealth app Did not use RE-AIM |
| Strategies for Implementing Occupational eMental Health Interventions: Scoping Review | Not original article |
| PRIMARY CARE CLINICIANS' IMPRESSIONS OF A PREVENTIVE GENOMICS CLINIC | Did not address the topic Did not use mHealth app |
| Points of Concordance, Points of Discordance: A Qualitative Examination of Telemedicine Implementation | Did not use mHealth app Did not use RE-AIM |
| Descriptive Analysis of a Telephone Based Community Monitoring Service for COVID-19 | Did not use mHealth app Did not use RE-AIM |
| Supporting population mental health and wellness during the COVID-19 pandemic in Canada: Protocol for a sequential mixed-method study | Did not use mHealth app Did not use RE-AIM |
| 77 Using Mobile Integrated Health and Telehealth to Support Transition of Care Amond Heart Failure Patients: Mighty Heart Study Protocol | Did not use mHealth app Did not use RE-AIM |
| Use of Video Telehealth Tablets to Increase Access for Veterans Experiencing Homelessness | Not original article |
| Implementation and Evaluation of the Virtual Graded Repetitive Arm Supplementary Program (GRASP) for Individuals With Stroke During the COVID-19 Pandemic and Beyond | Not original article |
| An implementation and longitudinal evaluation framework of remote quality improvement initiatives | Not original article |
| Scope: A new service supporting family doctors dealing with psychiatric patients in the community: Current utilization and quality improvement implementation protocol in the covid era | Study Protocol |
| Impact of non-pharmacological interventions in indigenous populations with diabetes mellitus on cardiovascular and kidney disease: A scoping review using the reaim framework | Not original article |
| mHealth for Integrated People-Centred Health Services in the Western Pacific: A Systematic Review | Not original article |
| Protocol paper: Stepped wedge cluster randomized trial translating the ABCS into optimizing cardiovascular care for people living with HIV | Did not use mHealth app Did not use RE-AIM |
| Protocol for the process and feasibility evaluations of a new model of primary care service delivery for managing pain and function in patients with knee osteoarthritis (PARTNER) using a mixed methods approach | Did not use mHealth app Did not use RE-AIM |
| Telerehabilitation for rural veterans: A qualitative assessment of barriers and facilitators to implementation | Did not use mHealth app Did not use RE-AIM |
| Stakeholder feedback at four ePRO-naïve healthcare institutions about the need, effectiveness, and barriers to usage of a fully EHR-integrated ePRO tool. | Did not use mHealth app Did not use RE-AIM |
| Hepatitis C virus screening and care: Complexity of implementation in primary care practices serving disadvantaged populations | Did not use mHealth app Did not use RE-AIM |
| Scale-up and dissemination of a school-based resistance training program: RE-AIM evaluation of impact | Did not use mHealth app Did not use RE-AIM |
| Implementation of Teledermatology: Theory and Practice | Systematic review |
| 588 Measuring implementation of store-and-forward teledermatology in Department of Veterans Affairs | Did not use mHealth app Did not use RE-AIM |
| Making connections: Nationwide implementation of video telehealth tablets to address access barriers in veterans | Did not use mHealth app Did not use RE-AIM |
| Using implementation facilitation to implement primary care mental health integration via clinical video telehealth in rural clinics: protocol for a hybrid type 2 cluster randomized stepped-wedge design | Did not use mHealth app Did not use RE-AIM |
| Electronic Consultation Services Worldwide: Environmental Scan | Not original article |
| Evaluating diverse electronic consultation programs with a common framework | Did not use mHealth app Did not use RE-AIM |
| Using the RE-AIM Framework in Formative Evaluation/Planning of a Mobile Prehospital Telestroke Intervention in an Urban Setting: Pilot Data for the Prehospital Rapid Evaluation via Ambulance Lead Emergency Remote Telemedicine (PRE-ALERT) Study | Did not use mHealth app Did not use RE-AIM |
| A school-based intervention incorporating smartphone technology to improve health-related fitness among adolescents: Rationale and study protocol for the NEAT and ATLAS 2.0 cluster randomised controlled trial and dissemination study | Study Protocol |
| A systematic review of eHealth cancer prevention and control interventions: New technology, same methods and designs? | Not original article |
| Web 2.0 chronic disease self-management for older adults: a systematic review. | Not original article |
| Assessing the internal and external validity of mobile health physical activity promotion interventions: a systematic literature review using the RE-AIM framework. | Not original article |
| Long-term results of a smoking reduction program | Did not address the topic Did not use mHealth app |
| Evaluating Initial Reach and Robustness of a Practical Randomized Trial of Smoking Reduction | Did not address the topic Did not use mHealth app |
| FoRSHE-X digital health intervention to improve the quality of life during chemotherapy among gynecological cancer survivors in Indonesia: A protocol for a pilot and feasibility study | Did not use mHealth app Did not use RE-AIM |
| Implementing a virtual emergency department to avoid unnecessary emergency department presentations | Did not use mHealth app Did not use RE-AIM |
| Implementation frameworks guiding digital self-management intervention in chronic pain: A scoping review | Not original article |
| Implementation of a home-based colorectal cancer screening intervention in Malaysia (CRC-SIM) | Did not use mHealth app Did not use RE-AIM |
| Nurse-to-family telehealth for pediatric transfers: protocol for a feasibility and pilot cluster randomized controlled trial | Did not use mHealth app Did not use RE-AIM |
| Virtual family-centered hospital rounds in the neonatal intensive care unit: protocol for a cluster randomized controlled trial | Did not use mHealth app Did not use RE-AIM |
| Low-Intensity mental health Support via a Telehealth Enabled Network for adults with diabetes (LISTEN): protocol for a hybrid type 1 effectiveness implementation trial | Study Protocol |
| Translation of a family-based behavioral intervention for adolescent obesity using the RE-AIM framework and common steps from adaptation frameworks | Study Protocol |
| Implementation Challenges for a Multisite Advance Care Planning Pragmatic Trial: Lessons Learned | Case Report |
| Evaluation of a physical activity and diet intervention delivered by telehealth for the secondary prevention of stroke: A Process evaluation of the ENAbLE Pilot Trial using the RE-AIM framework | Study Protocol |
| Assessing the Pragmatic Nature of Mobile Health Interventions Promoting Physical Activity: Systematic Review and Meta-analysis | Not original article |
| Keep it up! 3.0: Study protocol for a type III hybrid implementation-effectiveness cluster-randomized trial | Study Protocol |
| Implementing and Evaluating Community Health Worker-Led Cardiovascular Disease Risk Screening Intervention in Sub-Saharan Africa Communities: A Participatory Implementation Research Protocol | Did not use mHealth app Did not use RE-AIM |
| RE-AIM (Reach, Effectiveness, Adoption, Implementation, and Maintenance) Evaluation of the Use of Activity Trackers in the Clinical Care of Adults Diagnosed With a Chronic Disease: Integrative Systematic Review | Not original article |
| Implementing the Better Starts For All Pilot Mobile and Telehealth Intervention in Ohio Appalachia: Improving Access to Maternal Healthcare | Did not use mHealth app Did not use RE-AIM |
| Multi-method study of the implementation of Cognitive Symptom Management and Rehabilitation Training (CogSMART) in real-world settings | Did not use mHealth app Did not use RE-AIM |
| DIGITAL HEALTH INTERVENTIONS FOR PAIN IN PEDIATRIC ONCOLOGY: STATE OF THE FIELD | Not original article |
| Prehabilitation before gastrointestinal cancer surgery (PREHAB-GI): an implementation study | Did not use mHealth app Did not use RE-AIM |
| Adaptation and Dissemination of a National Cancer Institute HPV Vaccine Evidence-Based Cancer Control Program to the Social Media Messaging Environment | Did not use mHealth app Did not use RE-AIM |
| EFFECTIVENESS OF AN INTELLIGENT SLEEP MANAGEMENT SYSTEM IN THE US MILITARY: PRELIMINARY RESULTS | Did not use mHealth app Did not use RE-AIM |
| Reaching Older People With a Digital Fall Prevention Intervention in a Swedish Municipality Context-an Observational Study | Did not use RE-AIM |
| The NUDGE trial pragmatic trial to enhance cardiovascular medication adherence: study protocol for a randomized controlled trial | Study Protocol |
| Protocol for an Effectiveness-Implementation Hybrid Trial to Evaluate Scale up of an Evidence-Based Intervention Addressing Lifestyle Behaviours From the Start of Life: INFANT | Study Protocol |
| Optimising symptom management in children with cancer using a novel mobile phone application: protocol for a controlled hybrid effectiveness implementation trial (RESPONSE) | Did not use mHealth app Did not use RE-AIM |
| A cluster randomized controlled trial comparing Virtual Learning Collaborative and Technical Assistance strategies to implement an early palliative care program for patients with advanced cancer and their caregivers: a study protocol | Study Protocol |
| Internal and external validity of social media and mobile technology-driven HPV vaccination interventions: Systematic review using the reach, effectiveness, adoption, implementation, maintenance (RE-AIM) framework | Not original article |
| Implementation of early follow-up care after heart failure hospitalization | Did not use mHealth app Did not use RE-AIM |
| Implementation of Technology-Delivered Diabetes Self-care Interventions in Clinical Care: a Narrative Review | Not original article |
| Qualitative assessment of rapid system transformation to primary care video visits at an academic medical center | Did not address the topic Did not use RE-AIM |
| Sustainable support solutions for community-based rehabilitation workers in refugee camps: Piloting telehealth acceptability and implementation | Pilot study |
| Implementing internet-and tele-based interventions to prevent mental health disorders in farmers, foresters and gardeners (ImplementIT): Study protocol for the multi-level evaluation of a nationwide project | Study Protocol |
| Using the RE-AIM framework to evaluate internal and external validity of mobile phone-based interventions in diabetes self-management education and support | Not original article |
| Interactive digital health tools to engage patients and caregivers in discharge preparation: Implementation study | Study Protocol |
| Preventing diabetes with digital health and coaching for translation and scalability (PREDICTS): A type 1 hybrid effectiveness-implementation trial protocol | Study Protocol |
| iAmHealthy: Rationale, design and application of a family-based mHealth pediatric obesity intervention for rural children | Study Protocol |
| Digitally supported program for type 2 diabetes risk identification and risk reduction in real-world setting: protocol for the StopDia model and randomized controlled trial | Study Protocol |
| Mixed-methods approach to evaluate an mHealth intervention to increase adherence to triage of human papillomavirus-positive women who have performed self-collection (the ATICA study): Study protocol for a hybrid type i cluster randomized effectiveness-implementation trial | Did not use mHealth app Did not use RE-AIM |
| From evidence-based research to practice-based evidence: Disseminating a web-based computer-tailored workplace sitting intervention through a health promotion organisation | Did not use mHealth app Did not use RE-AIM |
| Online prevention aimed at lifestyle behaviors: a systematic review of reviews. | Not original article |
